# Fluorinated Benzofuran and Dihydrobenzofuran as Anti-Inflammatory and Potential Anticancer Agents

**DOI:** 10.3390/ijms241210399

**Published:** 2023-06-20

**Authors:** Abeer J. Ayoub, Ghewa A. El-Achkar, Sandra E. Ghayad, Layal Hariss, Razan H. Haidar, Leen M. Antar, Zahraa I. Mallah, Bassam Badran, René Grée, Ali Hachem, Eva Hamade, Aida Habib

**Affiliations:** 1 Department of Biochemistry and Molecular Genetics, Faculty of Medicine, American University of Beirut, Beirut 1107 2020, Lebanon; abeer.ayoub@liu.edu.lb (A.J.A.); gachkar@sgub.edu.lb (G.A.E.-A.); razan.haidar95@outlook.com (R.H.H.); zahraamallah@outlook.com (Z.I.M.); 2 Laboratory of Cancer Biology and Molecular Immunology, Faculty of Sciences I, Lebanese University, Hadath 1104, Lebanon; bassam.badran@ul.edu.lb (B.B.); eva.hamade@ul.edu.lb (E.H.); 3 Department of Biological Sciences, School of Arts and Sciences, Lebanese International University, Bekaa Campus, Bekaa 146404, Lebanon; 4 Faculty of Medicine, Saint George University of Beirut, Achrafieh, Beirut 1100-2807, Lebanon; 5 Department of Biology, Faculty of Sciences II, EDST, Lebanese University, Fanar 90656, Lebanon; sandra.ghayad@univ-amu.fr; 6 Center for CardioVascular and Nutrition Research (C2VN), INSERM 1263, INRAE 1260, Aix-Marseille University, 13385 Marseille, France; 7 Laboratory for Medicinal Chemistry and Natural Products, Faculty of Sciences I, PRASE-EDST, Lebanese University, Hadath 1104, Lebanon; layalharis@gmail.com (L.H.); ahachem@ul.edu.lb (A.H.); 8 Université de Rennes, CNRS, ISCR (Institut des Sciences Chimiques de Rennes), UMR 6226, 35000 Rennes, France; rene.gree@univ-rennes1.fr; 9 Department of Basic Medical Sciences, College of Medicine, QU Health, Qatar University, Doha 2713, Qatar

**Keywords:** benzofuran, inflammation, macrophage, HCT116 cells, prostaglandin E_2_, cyclooxygenase-2

## Abstract

Benzofuran and 2,3-dihydrobenzofuran scaffolds are heterocycles of high value in medicinal chemistry and drug synthesis. Targeting inflammation in cancer associated with chronic inflammation is a promising therapy. In the present study, we investigated the anti-inflammatory effects of fluorinated benzofuran and dihydrobenzofuran derivatives in macrophages and in the air pouch model of inflammation, as well as their anticancer effects in the human colorectal adenocarcinoma cell line HCT116. Six of the nine compounds suppressed lipopolysaccharide-stimulated inflammation by inhibiting the expression of cyclooxygenase-2 and nitric oxide synthase 2 and decreased the secretion of the tested inflammatory mediators. Their IC_50_ values ranged from 1.2 to 9.04 µM for interleukin-6; from 1.5 to 19.3 µM for Chemokine (C-C) Ligand 2; from 2.4 to 5.2 µM for nitric oxide; and from 1.1 to 20.5 µM for prostaglandin E_2_. Three novel synthesized benzofuran compounds significantly inhibited cyclooxygenase activity. Most of these compounds showed anti-inflammatory effects in the zymosan-induced air pouch model. Because inflammation may lead to tumorigenesis, we tested the effects of these compounds on the proliferation and apoptosis of HCT116. Two compounds with difluorine, bromine, and ester or carboxylic acid groups inhibited the proliferation by approximately 70%. Inhibition of the expression of the antiapoptotic protein Bcl-2 and concentration-dependent cleavage of PARP-1, as well as DNA fragmentation by approximately 80%, were described. Analysis of the structure–activity relationship suggested that the biological effects of benzofuran derivatives are enhanced in the presence of fluorine, bromine, hydroxyl, and/or carboxyl groups. In conclusion, the designed fluorinated benzofuran and dihydrobenzofuran derivatives are efficient anti-inflammatory agents, with a promising anticancer effect and a combinatory treatment in inflammation and tumorigenesis in cancer microenvironments.

## 1. Introduction

Inflammation is the body’s immune defense against offending agents that disrupt the integrity of cellular homeostasis, such as infectious agents (bacteria, virus, fungi) or tissue necrosis, to reinstate cellular homeostasis [1]. Failure to repair this disruption causes chronic inflammation that leads, in many cases, to cancer progression [2]. It has been suggested that chronic inflammation participates in tumor initiation (inducing DNA damage and chromosomal abnormalities), promotion (inducing clusters of malignant cells), and progression (inducing angiogenesis and metastasis) [3].

Colorectal cancer (CRC) can be associated with or is raised from chronic inflammation, such as in the case of patients with inflammatory bowel diseases (Crohn’s disease or ulcerative colitis) [4]. Inflammatory cytokine and mediator release in the surrounding microenvironment may promote cancer progression and metastasis. Thus, targeting inflammation in CRC patients may be a promising therapeutic approach. Large epidemiological studies have shown that non-steroidal anti-inflammatory drugs (NSAIDs) reduce the risk of CRC and decrease mortality by 30–40% [5]. Evidence was based on the chemoprotective effect of cyclooxygenase-2 (COX-2) selective inhibitors [6]. In addition, COX-2 is highly expressed in CRC and is associated with elevated levels of prostaglandin (PG) E_2_ [7]. PGE_2_ was shown to activate phosphatidylinositol-4,5-bisphosphate 3-kinase, the PI3K/Akt cell survival pathway. An in vivo study has shown that the sequestration of PGE_2_ by the administration of PGE_2_ monoclonal antibody in mice decreased the growth of the transplantable tumors [8]. Inflammatory mediators such as interleukin (IL)-6 can also mediate tumor proliferation by activating the antiapoptotic protein Bcl-2 [9]. Moreover, inducible nitric oxide (NO) synthase 2 (NOS2) had a significant association with poor survival in human cancer. It may be used as a predictive biomarker for cancer progression [10]. Thus, the combinatory drug that may have anti-inflammatory and anticancer effects could be a promising therapy for tumorigenesis [11].

Benzofuran and dihydrobenzofuran are key pharmacophores that are widespread in many natural products and bioactive compounds. Ailanthoidol, a natural benzofuran, exhibits anticancer, antiviral, immunosuppressive, antioxidant, and antifungal activities [12], whereas eurothiocin B, a representative example of 2,3-dihydrobenzofurans, is an α-glucosidase inhibitor [13]. Griseofulvin and its analogs are known as antiviral and anticancer agents [14,15], and Aurone also has anticancer activity [16]. The importance of fluorine in medicinal chemistry stands, inter alia, behind its ability to enhance drug permeability and bioavailability [17]. For this purpose, nine fluorinated benzofuran and dihydrobenzofuran derivatives were synthesized and tested for both anti-inflammatory and anticancer activities. In this present study, the anti-inflammatory effects of these derivatives were assessed by measuring the IL-6, NO, chemokine CCL2, and PGE_2_ formation and protein expressions of COX-2 and NOS2 in response to LPS-treated macrophages. In addition, the effects of these derivatives on COX-1 and COX-2 activities were also studied in the presence of arachidonic acid (the COX substrate). For the anticancer effect, HCT116 cells were treated with these derivatives and tested for their ability to inhibit the proliferation and induce apoptosis by testing the Bcl-2 and PARP-1 protein expression, as well as the percentage of DNA cleavage.

## 2. Results and Discussion

### 2.1. Effect of Inflammation on Macrophages

**Figure 1:**
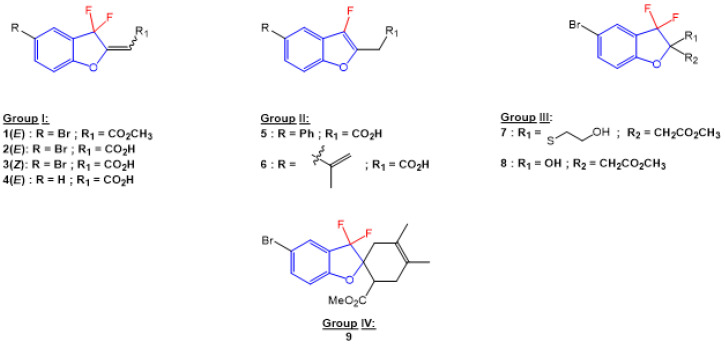
Structures of benzofuran and dihydrobenzofuran derivatives.

**Figure 2:**
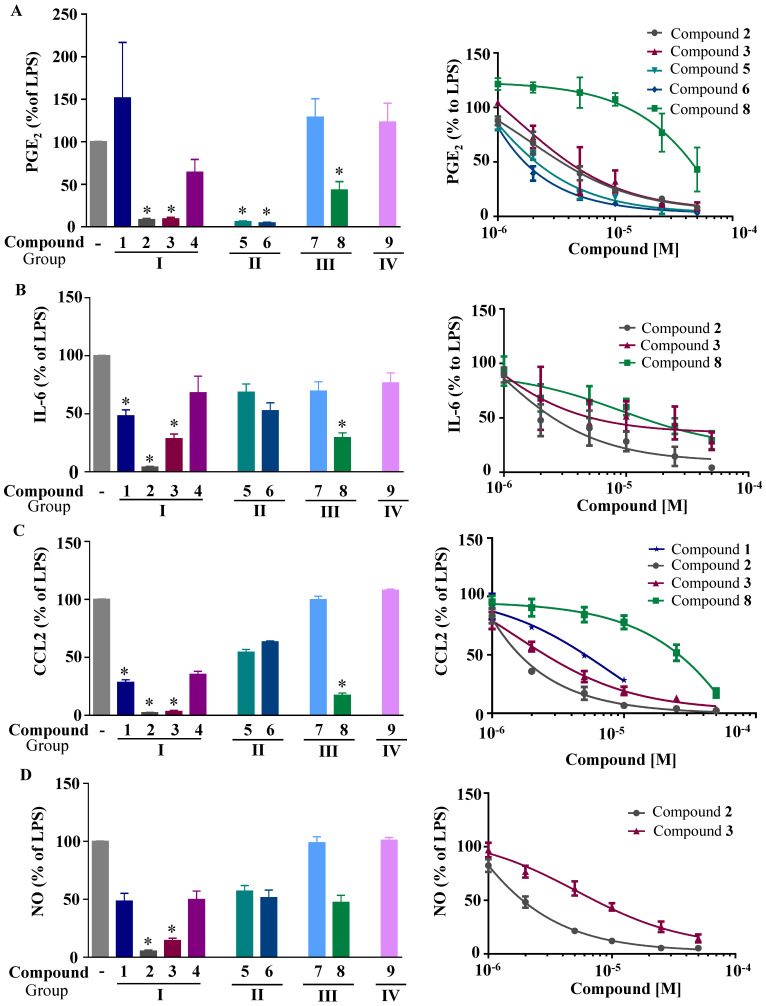
Effects of the fluorinated benzofuran and dihydrobenzofuran on the secretion of PGE_2_, IL-6, CCL2, and NO. Macrophages were treated with only 10 µM because of toxicity at higher concentrations for compounds **1**, **7**, and **9**, and 50 µM for the other compounds, prior to the addition of 10 ng/mL LPS for 24 h. The inflammatory mediators measured in the secreted milieu and results were expressed as percentage of LPS, and the corresponding IC_50_ fitting curves for compounds **2**, **3**, **5**, **6**, or **8** using six increasing concentrations were determined. (**A**) Secreted PGE_2_; (**B**) IL-6; (**C**) CCL2; and (**D**) NO formation. Levels of measured mediators and cytokines were for the basal and LPS-treated macrophages, as follows: PGE_2_: 19.9 ± 1.5 and 146 ± 27.2 pg/mL; IL-6: undetermined (for basal) and 19.9 ± 4.3 ng/mL; CCL2: 5.2 ± 0.4 and 54.7 ± 8.1 ng/mL; NO derivatives: 1.3 ± 0.1 µM and 31.4 ± 4.9 µM. Data are represented as mean of percentage of LPS ± SEM of four experiments performed in triplicates; * *p* < 0.05 versus LPS (one-way ANOVA followed by Dunnett’s test).

We first investigated the capacity of the fluorinated benzofuran and dihydrobenzofuran compounds on inflammation in LPS-treated macrophages. We assessed their inhibitory effects on the release of the proinflammatory mediators PGE_2_, IL-6, CCL2, and NO. For the difluorinated compounds of group I (Figure 1), we tested the role of the bromine and carboxyl group. Compounds **2** and **3** were very potent in blocking PGE_2_ formation by more than 50% production in response to LPS in macrophages, with IC_50_ values of 1.92 and 1.48 μM, respectively (Figure 2A and Table 1). For compounds of group II (Figure 1), which are monofluorinated, both compounds **5** and **6**, where R1 is the carboxyl group and the R is a phenyl group and an isopropyl group, respectively, were very potent inhibitors of PGE_2_ production in response to LPS in macrophages (Figure 2A). Finally, compound **8** of group III only showed a partial inhibition of PGE_2_ production, with an IC_50_ of 20.52 μM (Table 1).

Next, we evaluated the effect of the benzofuran derivatives on IL-6 and CCL2 in LPS-treated macrophages. Only compounds **2**, **3**, and **8** significantly decreased the IL-6 production by more than 50% at a concentration of 50 µM, with the IC_50_ ranging from 1.23 to 9.04 μM (Table 1, Figure 2B). For CCL2, compounds **2**, **3**, and **8** showed significant decreases (Figure 2C). Compound **1,** which was tested at its highest non-toxic concentration (10 µM), showed a moderate effect on CCL2. Compounds **1**, **2**, and to a lesser extent **3** and **4** were decreased significantly in the mRNA levels of CCL2 and IL-6 in human THP-1-derived macrophages treated with LPS (Figure S3).

In parallel, the effect of the synthesized compounds on the production of NO (Figure 2D) shows that only compounds **2** and **3** were able to significantly decrease NO production, with IC_50_ values of 2.4 and 5.2 µM, respectively.

**Figure 3:**
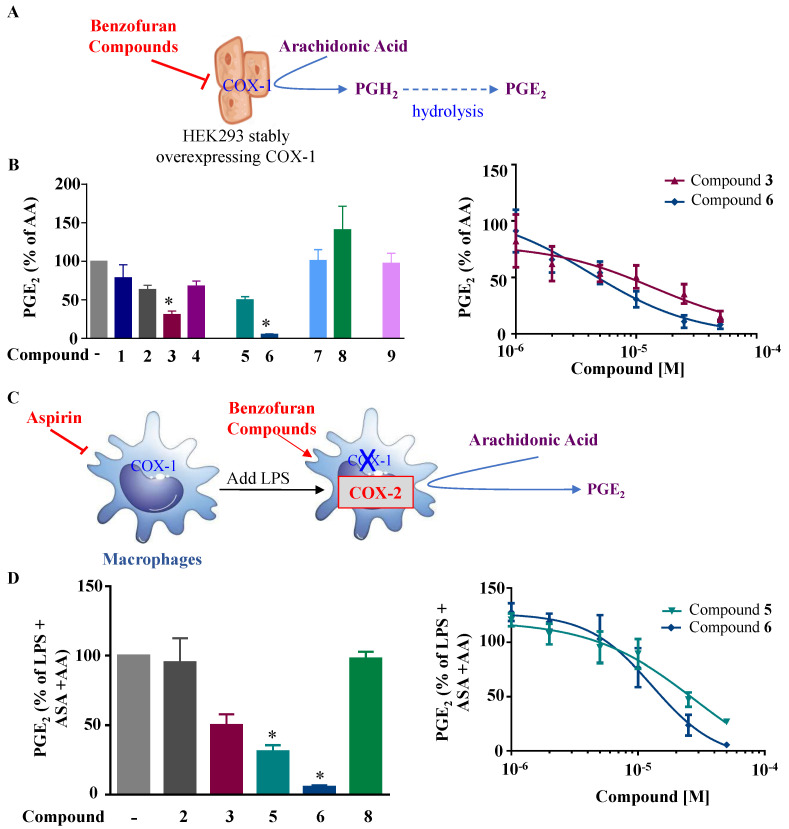
Effects of the fluorinated benzofuran and dihydrobenzofuran on COX-1 and COX-2 activities. (**A**) Illustration for COX-1 activity assay. HEK-293 cells stably overexpressing COX-1 are treated with the nine derivatives of benzofuran at concentrations of only 10 µM for compounds **1**, **7**, and **9**, and 50 µM for the other compounds, 30 min prior to the addition of 10 µM arachidonic acid (AA). This results in PGE_2_ production from PGH_2_ after hydrolysis, which reflects the COX-1 activity. (**B**) COX-1 activity. PGE_2_ formation was measured by enzyme immunoassay with the corresponding IC_50_ fitting curves for compounds **3** and **6**. Levels of PGE_2_ were very low in the absence of arachidonic acid (0.04 ± 0.01 ng/mL) and 104.3 ± 16.7 ng/mL for 10 µM AA. (**C**) Illustration for COX-2 activity determination. Macrophages are treated with 10 µM of aspirin (ASA) for 30 min to block basal COX activity, washed, and treated with 10 ng/mL LPS for 24 h to induce COX-2. Cells are then washed and incubated 30 min with 50 µM of the different compounds prior to the addition of 10 µM of AA. The produced PGE_2_ mainly reflects COX-2 activity. (**D**) COX-2 activity. Percentage of LPS-treated cells was calculated, and data are represented as mean ± SEM (n = 4); * *p* < 0.05 versus AA for COX-1 activity, and versus LPS + ASA + AA for COX-2 activity (one-way ANOVA followed by Dunnett’s test). Corresponding IC_50_ fitting curves for compounds **5** and **6** are illustrated. Levels of PGE_2_ were very low in the absence of arachidonic acid (0.06 ± 0.01 ng/mL) and 71.7 ± 5.7 ng/mL for 10 µM AA.

In order to determine whether these compounds have a direct effect on PGE_2_ formation, the COX activity and COX-2 expression were assessed. COX-1 activity was determined as the level of PGE_2_ synthesized from exogenous added arachidonic acid in HEK-293 cells overexpressing COX-1 (Figure 3A). In a similar setting, we previously showed that ibuprofen, a non-selective COX-1/COX-2 inhibitor, blocked PGE_2_ synthesis [18]. COX-2 activity was determined as the concentration of PGE_2_ synthesized from exogenous arachidonic acid added on 24 h LPS-treated macrophages, where previous treatment with aspirin was applied to block basal COX (Figure 3C). In this setting, 1 µM NS398, a selective COX-2 inhibitor (Cayman Chemicals, 70590), decreased PGE_2_ synthesis by 63% (data not shown). Figure 3B shows that compounds **3** and **6** significantly reduced the COX-1 activity, with IC_50_ values of 7.9 µM and 5 µM, respectively. In Figure 3D, we can see that compounds **5** and **6** strongly reduced the COX-2 activity, with IC_50_ values of 28.1 µM and 13 µM, respectively. Compound **3** showed a slight reduction in COX-2 activity. Compounds of group III (Figure 1) were generated from compound **1** and had bromine as the R group, with the ester group as R3, and R2 as the thiol with hydroxyl group for compound **7**, and only the hydroxyl group for compound **8**. Only compound **8** inhibited partial PGE_2_ production in macrophages (Figure 2A) without any effect of COX-1 or COX-2 activities (Figure 3B,D), whereas the replacement of R1 by the thiol–hydroxyl group (compound **7**) resulted in the loss of the inhibitory activity (Figure 2A and Figure 3B). Compound **9** did not have any effect (Figure 1A and Figure 3B).

**Figure 4:**
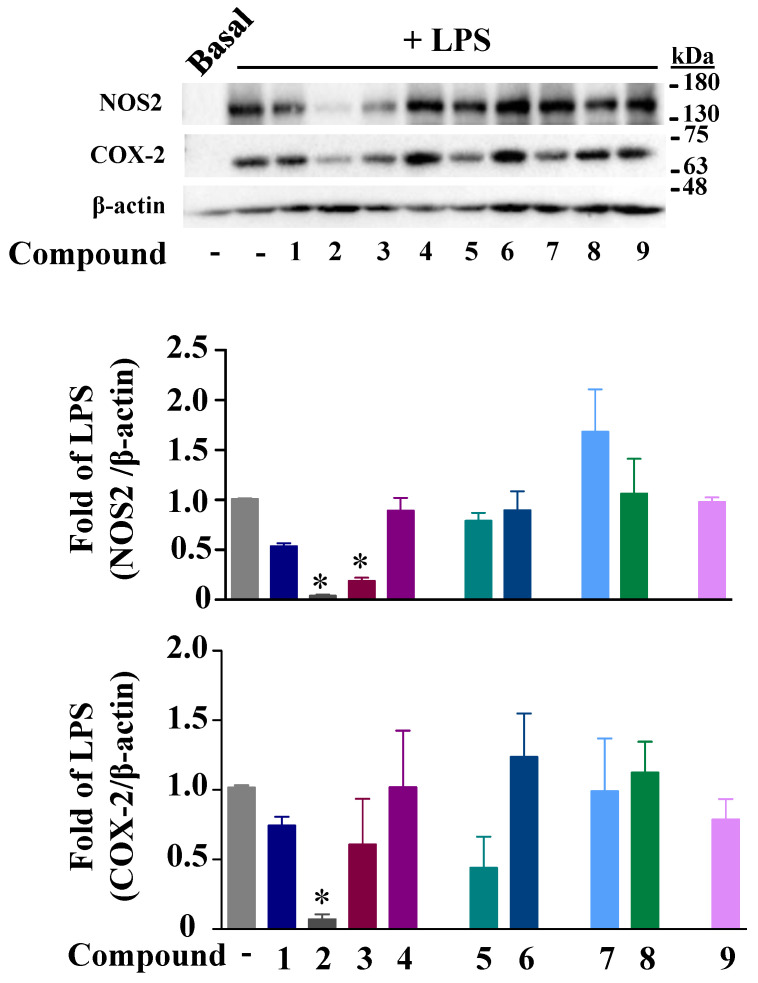
Effects of the fluorinated benzofuran and dihydrobenzofuran on COX-2 and NOS2 protein expressions. COX-2 and NOS2 protein expressions were assessed in macrophages incubated with only 10 µM for compounds **1**, **7**, and **9**, and 50 µM for the other compounds, for 30 min prior to the addition of 10 ng/mL LPS for 24 h. β-actin was used as a loading control, and densitometric analysis was performed using ImageJ software (NIH, MA). Data are expressed as fold of LPS-treated macrophages and represented as mean ± SEM of 4 different experiments; * *p* < 0.05 versus LPS (one-way ANOVA followed by Dunnett’s test).

In parallel, we assessed the effects of these compounds on the COX-2 and NOS2 expressions in macrophages. For the COX-2 expression, only compound **2** significantly decreased the COX-2 protein induced by LPS after 24 h of treatment (Figure 4). Replacing the R residue in compound **2** with hydrogen in compound **4** or R1 residue by a methyl ester group in compound **1** resulted in the loss of this inhibitory effect. Compounds **2** and **3** were the only compounds that significantly decreased the NO secretion. Because NO is the breakdown product of NOS2 enzymes, we assessed their effect on the enzyme expression. Figure 4 shows the inhibition of LPS-dependent NOS2 protein expression by compounds **2** and **3**. Compounds **1**, **2**, **3**, and **8** did not modify the NF-κB or Iκ-Bα phosphorylation induced by LPS, excluding any effects of the compounds on the NF-κB (Figure S4).

### 2.2. Effects of Compounds ***2***, ***3***, ***5***, and ***6*** on Inflammation in Subcutaneous Zymosan-Induced Air Pouch in Mice

**Figure 5:**
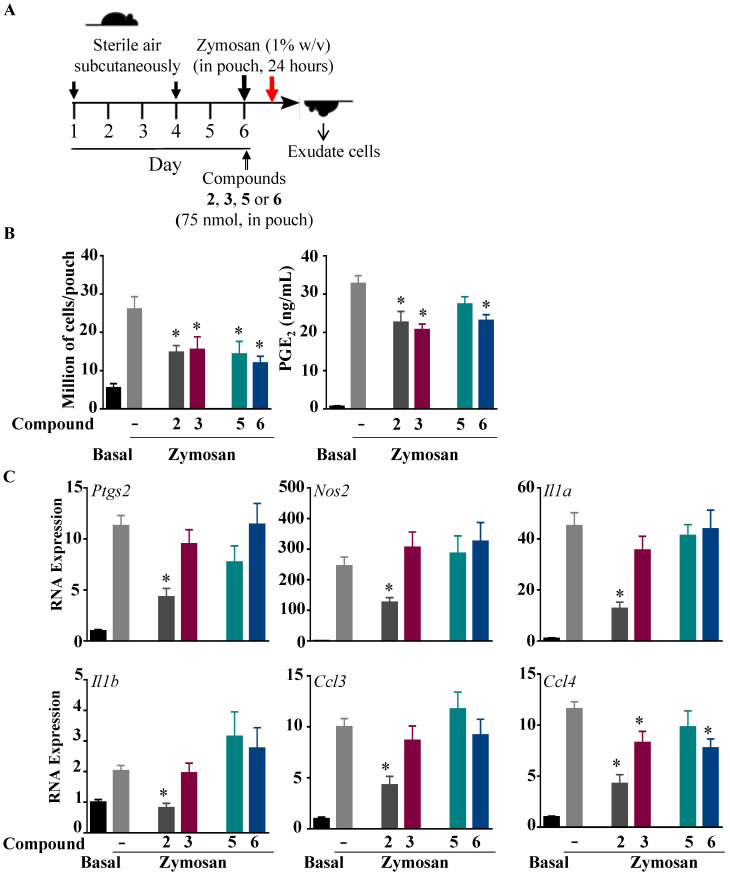
Effect of fluorinated benzofuran and dihydrobenzofuran on inflammation in the zymosan-treated sterile air pouch model in mice. (**A**) A timeline for the zymosan-induced inflammation experiments of the air pouch model in C57/BL6 mice. The pouch was formed on day 1 and refilled on day 4 with sterile air. Mice were injected at day 6 with 1% zymosan (*w*/*v* in saline), or 0.5 mL of saline for control mice. An amount of 50 nmol of compounds **2**, **3**, **5**, and **6**, or vehicle, were co-injected into the air pouch. At 24 h post-treatment, exudates were collected. (**B**) Recruited cells in the pouch and PGE_2_ production. (**C**) Gene expressions of inflammatory enzymes *Ptgs2* and *Nos2*, proinflammatory cytokines *Il1α* and *Il1β*, and chemokines *Ccl3* and *Ccl4*. Data correspond to the mean ± SEM (n = 6–8 mice per group); * *p* < 0.05 compared to the zymosan-treated cells (one-way ANOVA followed by Dunnett’s test).

The zymosan-injected air pouch model of inflammation has been shown to mimic the synovium cavity and is largely used to assess inhibitors of inflammation [19,20,21,22]. To test the anti-inflammatory effects of the synthesized compounds in vivo, compounds **2**, **3**, **5**, and **6** were co-administered in the sterile air pouch with zymosan (Figure 5A). At 24 h post-treatment, the number of cells of the air pouch exudates was significantly reduced for compounds **2**, **3**, **5**, and **6**, whereas the PGE_2_ levels were inhibited by compounds **2**, **3**, and **6** (Figure 5B). Compounds **2** and **6** inhibited LPS-induced IL-6 production, whereas compounds **3**, **5**, and **6** inhibited CCL2 formation in response to LPS, as assessed by ELISA (Figure S5). Next, we assessed the effects of these compounds on the gene expressions of several inflammatory biomarkers, including cytokines *Il1a* and *Il1b*, chemokines *Ccl3* and *Ccl4*, and the inflammatory-associated enzymes *Ptgs2* and *Nos2* (Figure 5C), and showed strong inhibition of the proinflammatory genes by compound **2**. Compounds **3** and **6** modestly inhibited the expression of *Ccl4*.

### 2.3. Anticancer Effect

**Figure 6:**
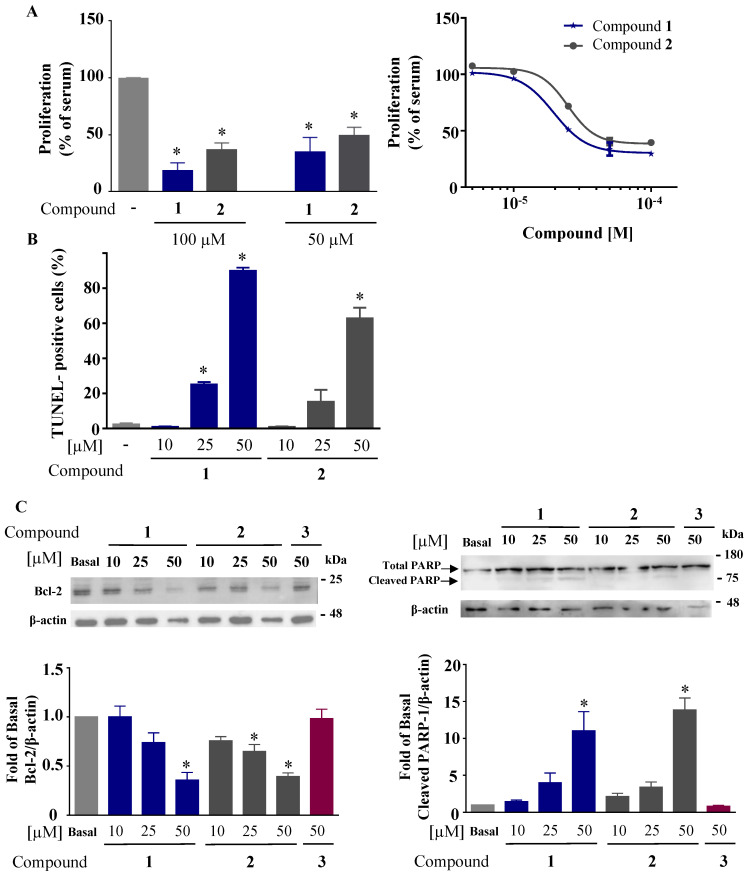
Effect of dihydrofluorinated benzofuran derivatives on HCT116 proliferation, DNA cleavage, PARP-1 cleavage, and Bcl-2 expression. (**A**) For proliferation, HCT116 cells were treated with 50 and 100 µM of all compounds for 72 h, and the percentage of viable cells was measured by WST-1 assay. The corresponding IC_50_ fitting curves for only the positive compounds **1** and **2**. (**B**) TUNEL assay for detecting the percentage of apoptotic cells (cleaved DNA) after treating with compounds **1** and **2** at 10, 25, and 50 µM for 72 h, compared to vehicle-treated cells showing no DNA cleavage (no treatment). (**C**) Western blot for Bcl-2 and cleaved PARP-1 after treatment of cells with compounds **1** and **2** at concentrations of 10, 25, and 50 µM for 72 h. Compound **3** was used as a negative control. β-actin was used as loading control. Data are represented as percentage mean ± SEM (n = 3); * *p* < 0.05 versus vehicle-treated cells (basal) (one-way ANOVA followed by Dunnett’s test).

Because studies have shown that some compounds with anti-inflammatory effects can have anticancerogenic effects, we evaluated the effect of the benzofuran derivatives on cell proliferation and apoptosis. The compounds were tested first for their antiproliferative effect on HCT116 using a WST-1 assay. Cells were treated with 50 and 100 µM of all the compounds for 72 h. Figure 6A shows that only compounds **1** and **2** inhibited cell proliferation by more than 50%, with IC_50_ values of 19.5 and 24.8 µM, respectively. A TUNEL assay was performed to assess the effect of these two compounds on apoptosis [23]. HCT116 cells were treated with compounds **1** and **2** at increasing concentrations (10, 25, and 50 µM) for 72 h. Figure 6B shows the concentration-dependent increase in the percentage of apoptotic cells for compounds **1** and **2**. Furthermore, the expression of the antiapoptotic protein Bcl-2 [24] was decreased by compounds **1** and **2** in a concentration-dependent manner in parallel to the increase in the protein level of cleaved PARP-1, a marker of apoptosis [25] (Figure 6C).

## 3. Materials and Methods

### 3.1. Materials

Arachidonic acid (90010), LPS (serotype 0111:B4, 19661), and PGE_2_-tracer (400140) for the PGE_2_ measurement were from Cayman Chemicals Co. (Ann Arbor, MI, USA), and ELISA kits for IL-6 and RT-PCR reagents were from Thermo Fisher Scientific (Waltham, MA, USA). All chemicals for the synthesis of the benzofuran derivatives, cell culture media and the WST-1 kit were from Sigma Aldrich (St. Louis, MO, USA), and all those for electrophoresis, protein quantification, and Western blot were from Bio-Rad Laboratories (Hercules, CA, USA)**.**

### 3.2. Synthesis of Fluorinated Benzofuran

Nine derivatives of benzofuran and dihydrobenzofuran with different structures were studied in this work (Figure 1).

The synthesis of compounds **1**, **7**, **8**, and **9** was described in our previous work [26]. Five novel derivatives, **2**, **3**, **4**, **5**, and **6**, were prepared by saponification of the corresponding esters (LiOH in THF/H_2_O and then acidification), purified by chromatography, and isolated with good yields (Table 2). NMR for the newly synthesized molecules is presented in the Supplementary Materials.

### 3.3. Evaluation of Inflammation in Bone Marrow-Derived Macrophages (BMDMs)

C57BL/6J (10–15 weeks old) male mice were obtained from the animal care facility at the American University of Beirut. All animal procedures were performed following the recommendations of the IACUC (Institutional Animal Care and Use Committee approval 16-09-M379). BMDMs were prepared and characterized as described previously by our group [27]. BMDMs were isolated from C57BL/6J mice by flushing the femur and tibia with RPMI-1640 culture media (Sigma-Aldrich R7388), and cells were collected and centrifuged at 300× *g* for 5 min. The supernatant was discarded, and the pellet was resuspended in 2 mL red blood lysis 10% (Sigma-Aldrich R7767) medium in PBS and incubated for 5 min at room temperature. After diluting the suspension of cells with 5 mL of RPMI containing 10% FBS and 1% penicillin–streptomycin, cells were centrifuged at 300× *g* for 5 min, and the cell pellet was resuspended in RPMI media containing 10% FBS supplemented with 15% L929-conditioned medium containing macrophage-colony stimulating factor (M-CSF1) and plated on 100 Petri dishes. Cells were allowed to proliferate and differentiate for 5–6 days. Cells were harvested, washed, and allowed to adhere overnight in RPMI containing 10% FBS for treatment. Cells were previously characterized by flow cytometry as macrophages using this method [28]. We used male C57BL/6J mice, as comparable responses to LPS were seen when using male or occasionally female mice (IL-6 levels varied from 4 to 20 ng/mL and from 6 to 20 ng/mL for female and male mice, respectively, and CCL2 levels varied from 1.5 to 5 ng/mL and from 2.1 to 7 ng/mL for female and male mice, respectively). Cells were plated at 0.8 × 10^6^ cells per 3.8 cm^2^ well [27].

### 3.4. Cell Treatment

BMDMs were then treated with different concentrations of the benzofuran and dihydrobenzofuran derivatives for 30 min prior to the addition of 10 ng/mL LPS for 24 h. Vehicle (dimethyl sulfoxide, DMSO) concentration did not exceed 0.4% and had no effect. The supernatants were assessed for IL-6, PGE_2_, CCL2, and nitrite, the stable derivative of NO, as described previously [19,28].

### 3.5. Cyclooxygenase Activity

COX-1 and COX-2 activities were performed as described previously [28] in human embryonic kidney cell line (HEK-293) stably overexpressing COX-1 [19] and in macrophages, respectively. Macrophages were first treated for 30 min with 10 μM acetylsalicylic acid (ASA) (Sigma-Aldrich, A5376) to irreversibly inhibit COXs and washed twice with PBS, prior to the addition of LPS for 24 h to induce COX-2. For both COX-1 and COX-2 assays, the benzofuran compounds were then added to the cells for 30 min prior to the incubation with 10 µM arachidonic acid for another 30 min. PGE_2_ was then measured using an enzyme immunoassay, as described previously [19,28].

### 3.6. Subcutaneous Dorsal Air Pouch Model

C57BL/6J female mice (8-week-old, 20–25 g) were kept at 5 per cage on a 12 h light–dark cycle with cotton cocoon as enrichment in temperature- and humidity-controlled rooms. Mice were provided with food and water *ad libitum*, and food intake and body weight were monitored throughout the study period. Approval for use of animals was obtained from the IACUC (16-11-393). Dorsal air pouches were created in mice as previously described [19,28]. Six days after the initial injection of sterile air, 0.5 mL of saline containing 100 µM of benzofuran compounds **2, 3**, **5**, or **6** (50 nmol per pouch) or 0.2% DMSO as vehicle was co-injected in the pouch with 0.5 mL of 0.9% saline (for the control group) or 0.5 mL of 1% zymosan (*w*/*v*, for zymosan and zymosan + compound) (Sigma-Aldrich, 400140). At 24 h after treatment, the animals were sacrificed by CO_2_ inhalation and the air pouch washed, the exudates collected, and the cell number determined. As previously reported, the main subtype of leukocytes present in the air pouch were neutrophils (75%) and monocytes–macrophages (12–15%), as determined by selective flow cytometry [20]. The concentrations of PGE_2,_ CCL2, and IL-6 and the gene expression were assessed as described previously [19,20,28].

### 3.7. Reverse Transcriptase-PCR (RT-PCR)

For gene expression, RT-PCR was performed as described previously [20]. Briefly, cell pellets obtained by centrifugation of air pouch exudates at 300× *g* for 5 min at 4 °C were suspended in TriPure (Sigma Aldrich 11667165001). An amount of 2 μg of total RNA was reverse-transcribed using iScript cDNA synthesis kit (Bio-Rad Laboratories, 1708891). RT-PCR was carried out on CFX384 cycler using iTaq Universal SYBR Green Supermix kit (Bio-Rad Laboratories, 1725121) and the primers obtained from TIB Molbiol (Berlin, Germany). Oligonucleotide sequences were according to the references [29,30,31], except for *Ptgs2* and *Nos2*, which were as follows: *Ptgs2* (F): AGACAGATTGCTGGCCGGGTTGCT; *Ptgs2* (R): TCAATGGAGGCCTTTGCCACTGCT; *Nos2* (F): CCCTTGTGCTGTTCTCAGCCCAAC; *Nos2* (R): GGACGGGTCGATGTCACATGCA. Gene expression was calculated using ^ΔΔCT^ method relative to the housekeeping gene 18S.

### 3.8. Toxicity Assay

WST-1 assay (Sigma-Aldrich, 5015944001) was used to determine the toxicity of the synthesized compounds on BMDM and HCT116 (ATCC, CCL-247), kindly provided by Dr. Nadine Darwiche, Department of Biochemistry and Molecular Genetics, American University of Beirut, Lebanon. Briefly, macrophages (50,000 cells per well) and HCT116 (20,000 cells per well) were plated in a 96-well plate in RPMI 1640 culture medium containing 10% fetal bovine serum (FBS) and grown for 24 h. Cells (in triplicates) were treated with 10 or 50 μM of the compounds for macrophages, and 50 or 100 μM for HCT116. Culture medium without cells and cells without treatment were used as basal and maximal activity, respectively. Results were expressed as percentage of cells without treatment. For macrophages, all compounds showed 95% viability at both concentrations except for compounds **1**, **7**, and **9**, with only 50% viability at 50 µM, which were further used at 10 µM (Figure S1). However, all compounds showed 95% viability at 100 µM in HCT116 (Figure S2).

### 3.9. Proliferation Assay

WST-1 assay was used to determine the effect of these compounds on HCT116 proliferation. Cells were plated in 96-well plate in triplicates (5000 cells per well) for 24 h and treated with 50 and 100 µM of the different compounds for 72 h. For compounds that exhibited strong antiproliferative effects, a dose–response curve was conducted with 5 concentrations (5, 10, 20, 50, and 100 µM). Culture medium without or with 10% FBS alone was used as basal and maximal proliferation, respectively. Results were expressed as percentage of cells incubated with 10% FBS alone.

### 3.10. Western Blot

Macrophages were incubated with the compounds for 30 min prior to the addition of 10 ng/mL LPS for 24 h. Protein levels for COX-2 and NOS2 were detected in LPS-stimulated macrophages. As previously described [28], 10 µg of total protein was assessed. The primary antibodies were developed and characterized as previously described: for COX-2, mouse monoclonal antibody anti-COX-2 (clone COX-214, 1/5000) [32]; for NOS2, rabbit polyclonal antibody anti-NOS2 (dilution1/2000 [33]). Antibodies anti-phospho-NF-κB (3033S), phospho-IκBα (9246S), and total IκBα (4814S) were from Cell Signaling Technologies (Danvers, MA, USA). Total NF-κB C-20 (sc-372) was from Santa Cruz Biotechnologies (Dallas, TX, USA), and mouse β-actin (dilution 1/10,000) (Sigma-Aldrich, A5441). Clarity™ western ECL substrate (Bio-Rad Laboratories 10-5061) was used according to the manufacturer’s instructions to reveal positive bands visualized using Bio-Rad ChemiDoc. For Bcl-2 and cleaved PARP-1, HCT116 cells were plated in 6-well plates (200,000 cells per well) for 24 h, and treated with compounds **1** and **2** at 10, 25, and 50 µM for 72 h. Supernatants and cells were collected and lysed using RIPA lysis buffer containing inhibitors of protease. Western blot of Bcl-2 and cleaved PARP-1 was performed using 25 µg of total protein with a rabbit polyclonal antibody anti-Bcl-2 (1/500) (Santa Cruz Biotechnology, sc-492), rabbit polyclonal antibody anti-PARP-1 (1/1000) (Santa Cruz Biotechnology, sc-7150), and mouse anti-β-actin (dilution 1/10,000) (Sigma-Aldrich, A5441).

### 3.11. TUNEL Assay

Terminal deoxynucleotidyl transferase dUTP nick-end labeling (TUNEL) assay (Sigma Aldrich 11684795910) was conducted to detect late phase of apoptosis in HCT116 cells. Cells were plated in 6-well plate (200,000 cells per well) for 24 h, and treated with compounds **1** and **2** at 10, 25, and 50 µM for 72 h. Adherent cells and cells in supernatants were collected and fixed with 4% paraformaldehyde for 2 days and then treated with PBS containing 0.1% Triton X-100 at 4 °C for 2 min. Cells were then stained with the TUNEL reagent for 1 h, and positive signals were quantified using the Guava EasyCyte8 Flow Cytometer-Millipore according to the manufacturer’s instructions.

### 3.12. Data Analysis

IL-6, CCL2, nitrite (for NO), and PGE_2_ concentrations from 3–5 independent experiments were expressed as percentage of LPS alone and as mean ± SEM. COX activity was determined as percentage of PGE_2_ produced from exogenous arachidonic acid. Curve fitting and calculation of IC_50_ values were performed using GraphPad Prism (version 9.5.1 Software, La Jolla, CA, USA).

Densitometric analyses were performed using ImageJ software (NIH, MA, USA). The ratio of the different analyzed bands to β-actin was determined and data are represented as fold change compared to the LPS-treated cells. Percentage of viable cells for proliferation assay was determined as percentage to control (non-treated cells). For TUNEL assay, apoptotic cells were quantified, and results were expressed as percentage to positive cells (cleaved DNA) compared to control (non-treated cells). Statistical analysis was performed using one-way ANOVA, followed by Dunnett’s multiple comparisons test using GraphPad Prism software. Differences were considered significant when *p* < 0.05.

## 4. Conclusions

Benzofuran derivatives were shown to have important pharmacological activities, including anticancer, antibacterial, antifungal, analgesic, and many others [34]. Adding fluorine molecules to benzofuran can enhance its bioavailability and permeability [35]. For this, we used nine newly synthesized dihydrofluorinated benzofuran derivatives and aimed to find out which compounds have a dual effect: anti-inflammatory and anticancer, with a potential application in the types of cancer associated with chronic inflammation, such as CRC. The reason behind this is the link between inflammatory mediators and cancer progression. For example, it was found that IL-6 promotes tumor progression by activating the MAPK pathway, which is known to induce tumor proliferation [16]. Others have shown that the chemokine CCL2 induces tumor proliferation and migration by activating the Akt pathway and matrix metalloproteases [36]. For COX-2, several studies have shown its implication in CRC progression, as its protein expression was significantly increased, especially in the latest stages of cancer. The inhibition of COX-2 expression or activity was also demonstrated to lower tumor progression in vivo [37]. One of the mechanisms might be the decrease in Bcl-2 expression, as it was shown that PGE_2_ can activate Bcl-2 expression; thus, by inhibiting COX-2, PGE_2_ is decreased, and thus Bcl-2 will also decrease, leading to tumor regression [38].

All these findings prompt us to synthesize combinatory molecules working on inhibiting inflammatory mediators, as well as on targeting cancer progression and inducing apoptosis. In our study, six out of nine compounds showed important anti-inflammatory effects with different efficiencies (**1**, **2**, **3**, **5**, **6**, and **8**) on PGE_2_, IL-6, CCL2, NO, and COX-1/2 activities, and on the COX-2 and NOS2 expressions. Among these six molecules, two were shown to have important anticancer effects (**1** and **2**) by inhibiting HCT116 proliferation, inducing PARP-1 and DNA cleavages, in parallel with a decrease in Bcl-2 expression. These compounds are difluorinated, having bromine as the R group, and with either formic acid (carboxylic acid group) for compound **1** or ethanoate (ester group) for compound **2** for the R1 group. Compounds **1**, **2**, **3**, and **8** have bromine as the R group and different groups at R1 and R2, which may support the difference in the efficiency of each molecule. Compound **4**, which lacks any of the biological effects tested, has a similar structure to compound **2** except for the bromine; thus, for benzofuran derivatives, bromine is important for the anti-inflammatory effect. Compounds **7** and **9** shared the same structure as compounds **1**, **2**, **3**, and **8**, but with differences in the R1 and R2, which were bulky and may stand behind their negative effects. Finally, compounds **5** and **6**, monofluorinated benzofuran derivatives with R groups other than bromine, such as in compounds **3** and **2**, showed a direct inhibitory effect on both COX-1 and COX-2 activities and no effect of COX-2 or NOS2 expression. This suggests that monofluorination is necessary for the inhibition of COX activities.

Overall, all these results showed the importance of some structural groups of benzofuran, mainly bromine and fluorine, on anti-inflammatory and antiproliferative effects. In the present exploratory study, we identified some novel benzofuran molecules with the desired dual biological action (i.e., anti-inflammatory and anticancer). Compounds **1** and **2** are promising. In the near future, we aim to improve such properties and, based on molecular modeling studies, use these compounds in order to design improved analogues to be synthetized.

## Figures and Tables

**Table 1:** In vitro effects of compounds **1**, **2**, **3**, **5**, **6**, and **8** on the release of inflammatory mediators in macrophages.

Compound	IC_50_ (µM)			
	PGE_2_	IL-6	CCL2	NO
**1**	ND	ND	8	ND
**2**	1.91	1.23	1.52	2.42
**3**	1.48	5.21	1.5	5.23
**5**	1.92	ND	ND	ND
**6**	1.12	ND	ND	ND
**8**	20.52	9.04	19.27	ND

**Table 2:** Synthesis and yield of carboxylic acids **2**, **3**, **4**, **5**, and **6**.

Compound	Starting Compound	Yield
**2**	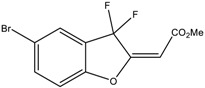	63%
**3**	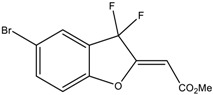	70%
**4**	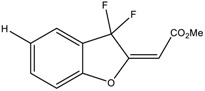	78%
**5**	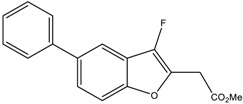	68%
**6**	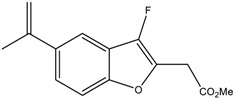	71%

## Data Availability

The data used to support the findings of this study are available from the corresponding author upon request.

## Supplementary Materials

The following supporting information can be downloaded at https://www.mdpi.com/article/10.3390/ijms241210399/s1.

## Abbreviations

AA: arachidonic acid; ASA: acetylsalicylic acid; Bcl-2: B-cell lymphoma 2; BMDMs: bone marrow-derived macrophages; CCL2: C-C Motif Chemokine Ligand 2; COX: cyclooxygenase; CRC: colorectal cancer; FBS: fetal bovine serum; IL-6: interleukin-6; LPS: lipopolysaccharide; MAPK: mitogen-activated protein kinase; NO: nitric oxide; NOS2: nitric oxide synthase 2; NSAIDs: non-steroidal anti-inflammatory drugs; PARP-1: Poly (ADP-Ribose) Polymerase 1; PGE_2_: prostaglandin E_2_; TUNEL: terminal deoxynucleotidyl transferase dUTP nick-end labeling.
