# Supplementary material for: Fluorinated Benzofuran and Dihydrobenzofuran as Anti-Inflammatory and Potential Anticancer Agents
Source: Int J Mol Sci. 2023 Jun 20;24(12):10399. doi: 10.3390/ijms241210399 (PMC10299677; doi:10.3390/ijms241210399)
Supplement: Supplementary file 1 [file ijms-24-10399-s001.zip › ijms-2433788-supplementary.pdf]

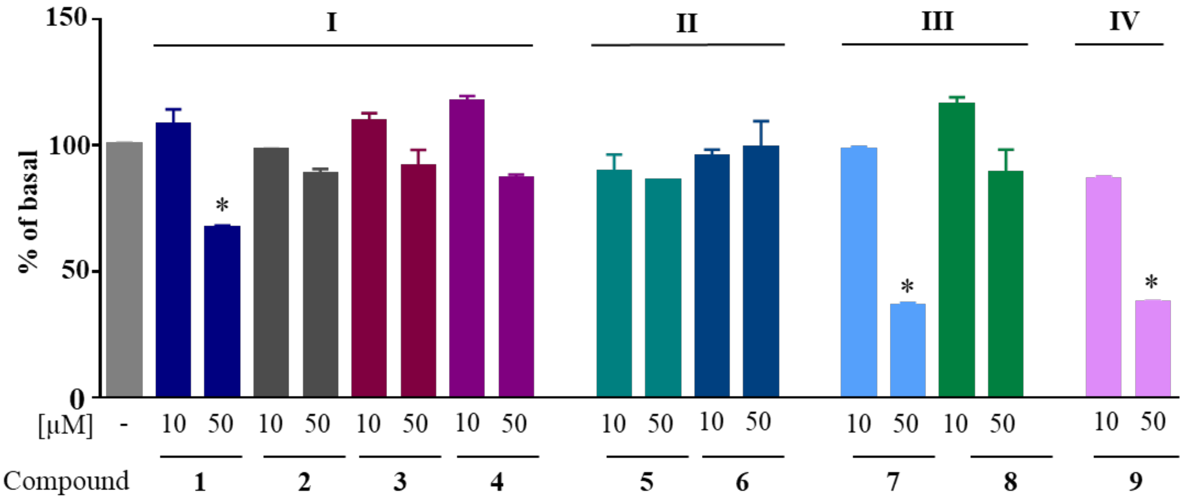

Figure S1: Effect of fluorinated benzofuran derivatives on macrophages viability. Cells incubated with 10 and 50 μM of benzofuran derivatives (compounds **1** to **9**) belonging to groups I, II, III and IV for 24 hours. Cell viability was determined by WST-1 assay. Non-treated macrophages were used as control. Results were shown as mean ± SEM (n=3). *p< 0.05 compared to the vehicle –treated cells (basal), one-way ANOVA followed by Dunnett's test.

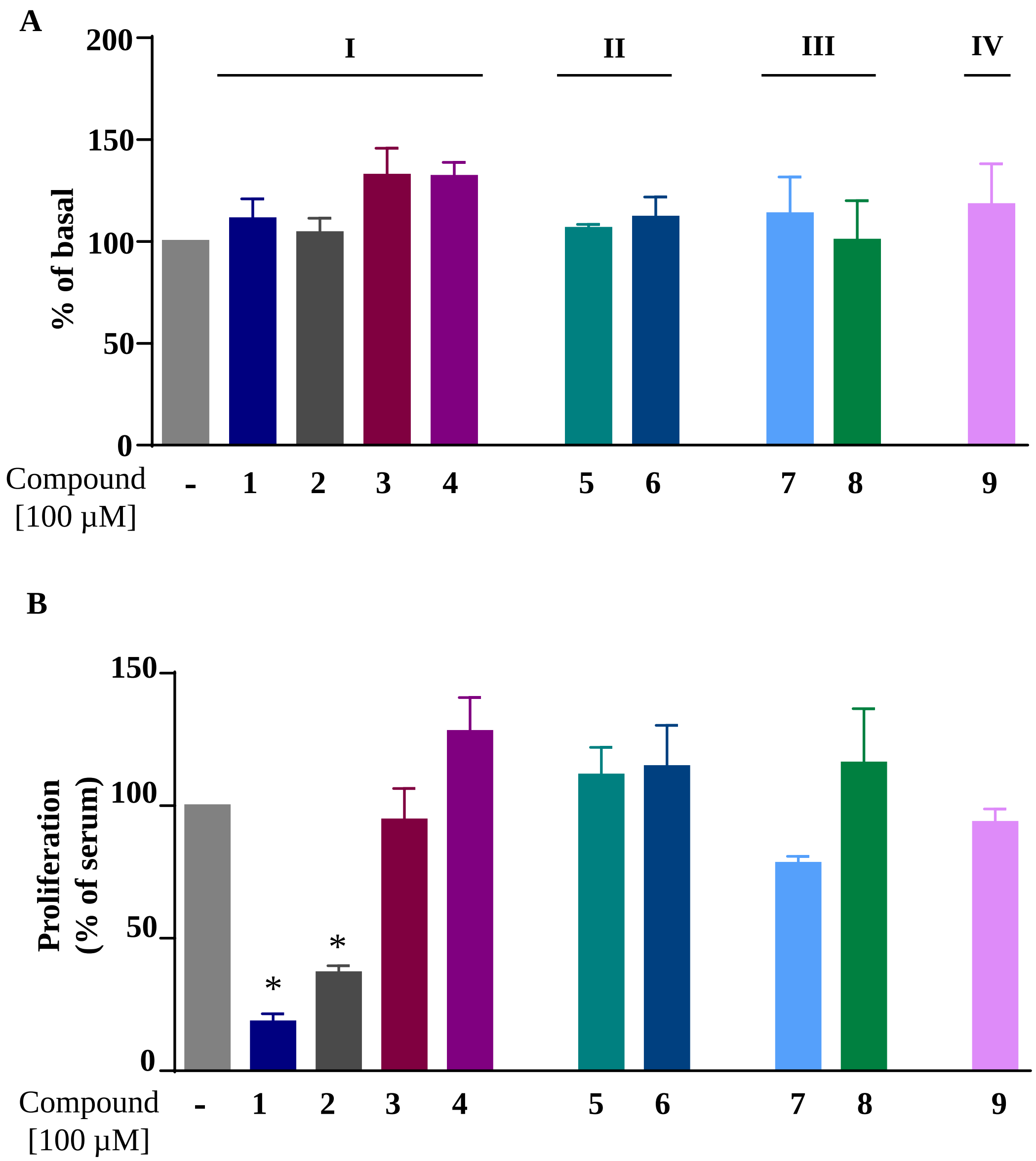

Figure S2: (A) Cytotoxicity of fluorinated benzofuran and dihydrobenzofuran compounds on HCT116. Cells were treated with 100 µM of each compound for 24 hours. Cytotoxicity was assessed by WST-1 assay. HCT116 cells treated with the vehicle corresponded to the basal. Percentage of basal are calculated and results are expressed as mean ± SEM (n=3); (**B)** Effect of dihydro-fluorinated benzofuran derivatives on HCT116 proliferation. HCT116 were treated with 100 µM of all compounds for 72 hours and the percentage of cells of untreated proliferative cells was determined by WST-1 assay. Data are represented as mean ± SEM (n=3), *$p<0.05$ versus vehicle-treated cells (Basal) (One-way Anova followed by the Dunnett's test).

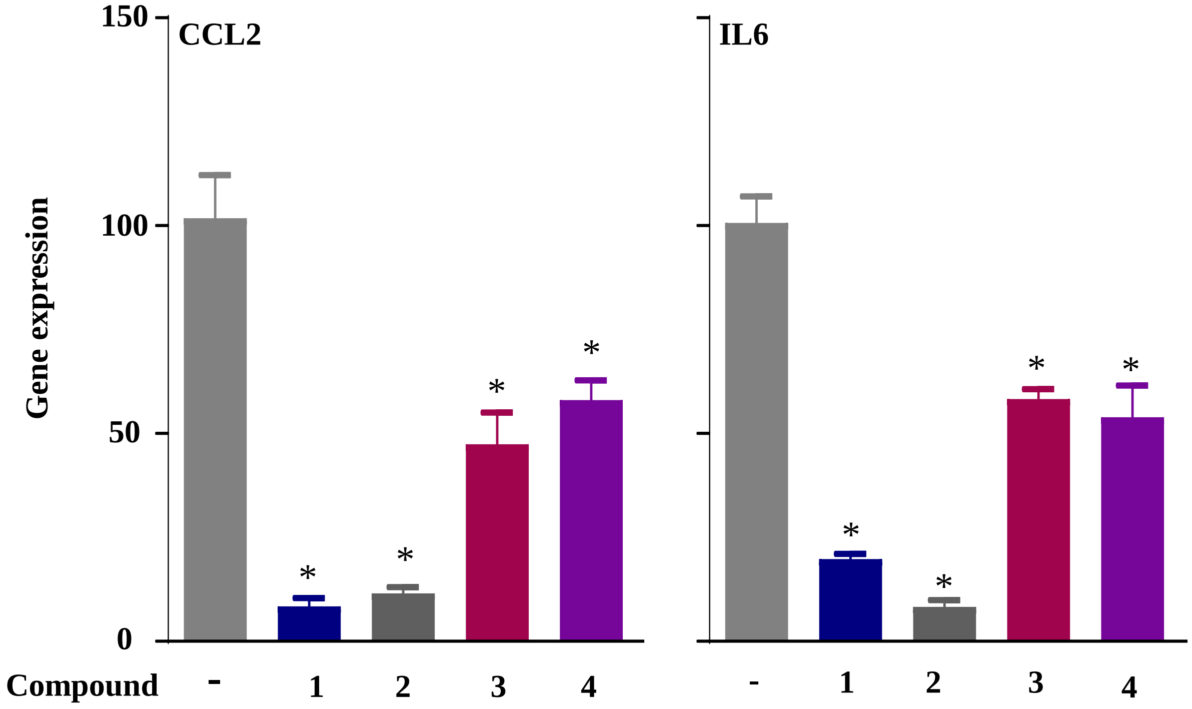

Figure S3: Effect of benzofuran compounds on the levels of LPS-dependent mRNA of CCL2 and IL-6 in THP-1 –derived macrophages. Human-monocytic cell line-THP-1 were treated with PMA (25 nM for 48 hours) then incubated with 50 µM compounds for 30 minutes before the addition of 10 ng/ml LPS for 6 hours. Gene expression of IL6 and CCL2 were measured by RT-PCR and the values were normalized to GAPDH. Results represent means ± SEM by one way ANOVA followed by Dunnett test.

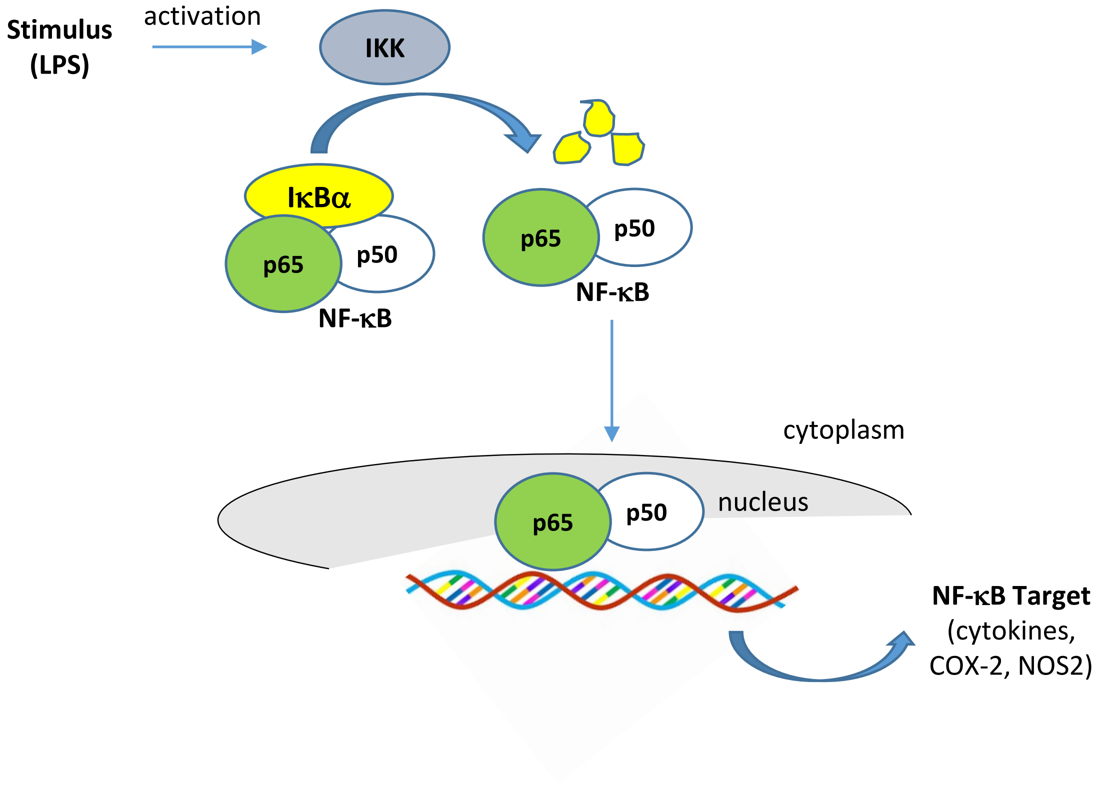

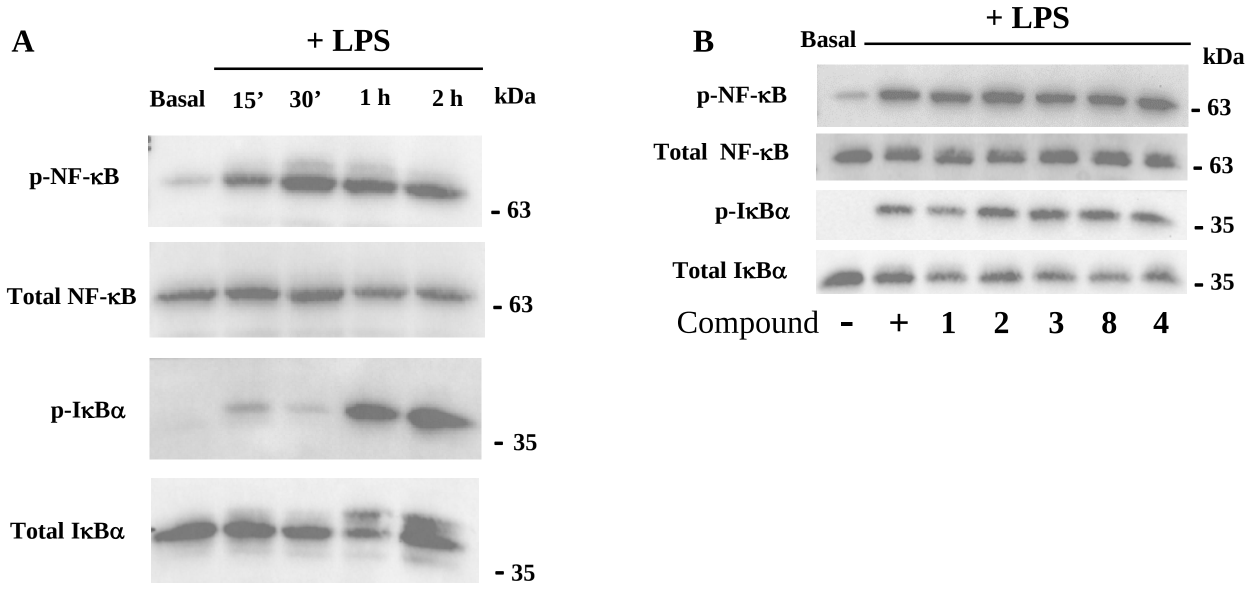

Figure S4: Phosphorylation of NF-κB and IκBα kinetics in LPS-treated macrophages. **(A)** Kinetics of the phosphorylation of NF-κB and IκBα in macrophages. Cells were treated with 10 ng/mL LPS for different time points and protein expression was assessed by western blot. We determine that 1 hour incubation with LPS is efficient for the phosphorylation of NF-κB and IκBα phosphorylation; **(B)** Macrophages were treated with the tested compounds for 30 minutes prior to 1 hour-treatment with LPS. β-actin was used as a loading control and densitometric analysis was done ImageJ Software (NIH, MA). Data are expressed as fold of LPS-treated macrophages and represented as mean ± S.E.M. of 3 different experiments, *$p<0.05$ versus LPS (One-way Anova followed by the Dunnett's test).

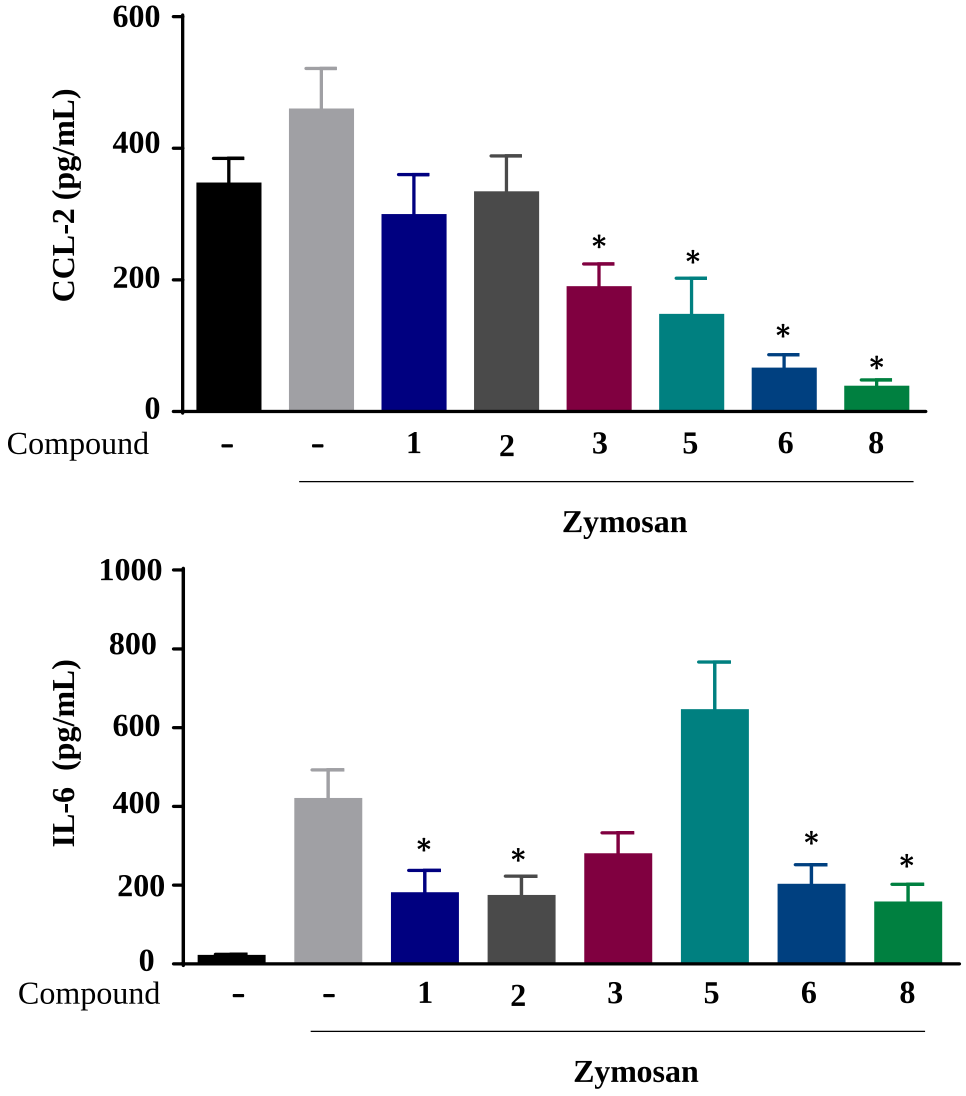

Figure S5: Effect of fluorinated benzofuran and dihydro-benzofuran on inflammation in the zymosan-treated sterile air pouch model in mice. CCL2 and IL-6 production in the air pouch was measured by ELISA. Data corresponds to the mean ± S.E.M. (n= 6-8 mice per group); *p< 0.05 compared to the zymosan -treated cells (one-way ANOVA followed by Dunnett’s test).

SUPPLEMENTAL MATERIAL

General information

The reaction mixtures were magnetically stirred with Teflon stirring bars. They were monitored by thin-layer chromatography (TLC), carried out on 0.25 mm Merck silica gel plates (60 F254) with detection by UV light or staining with *p*-anisaldehyde. The eluents used were mixtures of petroleum ether and ethyl acetate (EtOAc). Sigma Aldrich silica gel (60 Å, particle size 0.040–0.063 mm) was used for column chromatography. Nuclear magnetic resonance (NMR) spectra have been recorded with Bruker Avance 500, and 300 spectrometers. ^{1}H NMR spectra: δ (H) are given in ppm relative to tetramethylsilane (TMS), using [δ (C*H*Cl_3) = 7.26 ppm], or [δ (CH_3COCH_3) = 2.05 ppm] as internal reference. ^{13}C NMR spectra: δ (C) are given in ppm relative to TMS, using [δ (*C*DCl_3) = 77.0 ppm], or [δ (CD_3COCD_3) = 206.7, 29.9 ppm] as internal reference. ^{19}F NMR spectra: δ (F) are given in ppm relative to C*F*Cl_3 = 0.0 ppm as external reference. Multiplicities were designated as singlet (s), doublet (d), triplet (t), multiplet (m). Mass spectral analyses have been performed at the Centre Régional de Mesures Physiques de l'Ouest (CRMPO) in Rennes (France). The melting points were determined using a Kofler Hot Bench.

General procedure for the synthesis of carboxylic acids 2, 4 and 6

To a solution of ester **1, 3** or **5** in THF was added dropwise under magnetic stirring at room temperature, a solution of LiOH 2M until disappearance of the starting material (for 2h). The reaction mixture was filtered and the filtrate was extracted with ethyl acetate. The combined organic phases were dried over Na_2SO_4, filtered and concentrated in vacuum. Carboxylic acids were obtained after purification by chromatography on silica gel.

Synthesis of (*E*)-2-(5-bromo-3,3-difluorobenzofuran-2(3H)-ylidene)acetic acid 2(E)

The reaction was performed with ester (50 mg, 0.16 mmol; in 1 mL THF) and 2 mL LiOH according to the general procedure. After purification by chromatography on silica gel, acid **2(E)** was obtained as a white solid (30 mg, 63% yield).

***R*f** = 0.58 (petroleum ether-EtOAc; 6:4); **m.p.** = 150-152°C.

^{1}H NMR (CD_3COCD_3, 300 MHz): δ = 7.93-7.91 (m, 1H), 7.83-7.80 (m, 1H), 7.28-7.23 (m, 1H), 6.18 (t, 1H, *J* = 3.4 Hz).**^{13}C NMR (CD_3COCD_3, 75 MHz):** δ = 165.1 (t, $^{4}J_{C-F}$ = 2.2 Hz), 161.6 (t, $^{2}J_{C-F}$ = 29.1 Hz), 157.4 (t, $^{3}J_{C-F}$ = 7.0 Hz), 139.3 (t, $^{3}J_{C-F}$ = 1.2 Hz), 129.2, 124.5 (t, $^{2}J_{CF}$ = 26.2 Hz), 119.5 (t, $^{1}J_{C-F}$ = 247.2 Hz), 117.8 (t, $^{4}J_{CF}$ = 1.8 Hz), 115.7, 106.2 (t, $^{3}J_{C-F}$ = 1.2 Hz). **^{19}F NMR ($CDCl_3$, 282 MHz):** δ = -90.23 (s). **HRMS (ESI):** calcd. for $C_{10}H_6O_3F_2{}^{79}BrNa$: m/z $[M+Na]^+$ 312.92823; found: 312.9282 (0 ppm); calcd. for $C_{10}H_4O_3F_2{}^{79}BrNa_2$: m/z $[M-H+2Na]^+$ 334.91018; found: 334.9101 (0 ppm).

Synthesis of (*Z*)-2-(5-bromo-3,3-difluorobenzofuran-2(3H)-ylidene)acetic acid 3(Z)

The reaction was performed with ester (50 mg, 0.16 mmol) and 2 mL LiOH according to the general procedure. After purification by chromatography on silica gel, acid **3(Z)** was obtained as a white solid (33 mg, 70% yield).

***R*f** = 0.56 (petroleum ether-EtOAc; 6:4); **m.p.** = 140-142°C.

^{1}H NMR (CD_3COCD_3, 300 MHz): δ = 8.02-8.00 (m, 1H), 7.90-7.86 (m, 1H), 7.37-7.31 (m, 1H), 5.93 (t, 1H, *J* = 3.1 Hz). **^{13}C NMR (CD_3COCD_3, 75 MHz):** δ = 165.0 (t, $^{4}J_{C-F}$ = 2.6 Hz), 159.8 (t, $^{2}J_{C-F}$ = 27.4 Hz), 158.7 (t, $^{3}J_{C-F}$ = 7.5 Hz), 139.8 (t, $^{3}J_{C-F}$ = 1.2 Hz), 129.3, 122.6 (t, $^{2}J_{C-F}$

= 25.2 Hz), 120.8 (t, $^1J_{C-F}$ = 244.7 Hz), 118.1 (t, $^4J_{CF}$ = 1.6 Hz), 116.2, 101.2. **^{19}F NMR ($CDCl_3$, 282 MHz):** δ = -86.02 (s). **HRMS (ESI):** calcd. for $C_{10}H_5O_3F_2{}^{79}BrNa$: m/z $[M+Na]^+$ 312.92823; found: 312.9284 (0 ppm); calcd. for $C_{10}H_4O_3F_2{}^{79}BrNa_2$: m/z $[M-H+2Na]^+$ 334.91018; found: 334.9102 (0 ppm).

Synthesis of (*E*)-2-(3,3-difluorobenzofuran-2(3H)-ylidene)acetic acid 4(E)

The reaction was performed with ester (11 mg, 0.05 mmol) and 0.5 mL LiOH according to the general procedure. After purification by chromatography on silica gel, acid **4(E)** was obtained as a white solid (8 mg, 78% yield).

***R*f** = 0.31 (petroleum ether-EtOAc; 7:3); **m.p.** = 132-134°C.

^{1}H NMR (CD_3COCD_3, 500 MHz): δ = 7.73-7.76 (m, 1H), 7.69-7.65 (m, 1H), 7.36-7.32 (m, 1H), 7.26-7.23 (m, 1H), 6.14 (t, 1H, *J* = 3.3 Hz).**^{13}C NMR (CD_3COCD_3, 125 MHz):** δ = 165.3, 162.1 (t, $^2J_{C-F}$ = 29.6 Hz), 158.3 (t, $^3J_{C-F}$ = 7.3 Hz), 136.5, 126.4 (t, $^3J_{C-F}$ = 1.3 Hz), 126.3, 122.4 (t, $^2J_{C-F}$ = 26.0 Hz), 120.2 (t, $^1J_{C-F}$ = 245.8 Hz), 113.5, 105.3. **^{19}F NMR (CD_3COCD_3, 470 MHz):** δ = -89.93 (s). **HRMS (ESI):** calcd. for $C_{10}H_6O_3F_2Na$: m/z $[M+Na]^+$ 235.01772; found: 235.0177 (0 ppm); calcd. for $C_{10}H_6O_3F_2K$: m/z $[M+K]^+$ 250.99166; found: 250.9912 (2 ppm); calcd. for $C_{10}H_5O_3F_2Na_2$: m/z $[M-H+2Na]^+$ 256.99967; found: 256.9994 (1 ppm); calcd. for $C_{10}H_5O_3FNa$: m/z $[M-HF+Na]^+$ 215.01149; found: 215.0114 (0 ppm).

Synthesis of 2-(3-fluoro-5-phenylbenzofuran-2-yl)acetic acid 5

The reaction was performed with ester (30 mg, 0.10 mmol) and 1 mL LiOH according to the general procedure. After purification by chromatography on silica gel, acid **5** was obtained as a white solid (18 mg, 68% yield).

***R*f** = 0.25 (petroleum ether-EtOAc; 6:4); **m.p. =** 84-88 ^{o}C.

^{1}H NMR ($CDCl_3$, 300 MHz): δ = 7.74 (m, 1H), 7.63-7.62 (m, 1H), 7.60 (m, 1H), 7.54-7.53 (m, 1H), 7.48-7.46 (m, 2H), 7.45-7.44 (m, 1H), 7.39-7.36 (m, 1H),3.93 (d, 2H, *J* = 1.7 Hz). **^{13}C NMR ($CDCl_3$, 75 MHz):** δ = 172.7 (d, $^4J_{C-F}$ = 2.6 Hz), 151.3 (d, $^3J_{C-F}$ = 8.8 Hz), 146.2 (d, $^1J_{C-F}$ = 254.2 Hz), 141.0, 137.0, 133.4 (d, $^2J_{C-F}$ = 27.3 Hz), 128.8 (2C), 127.4 (2C), 127.2, 125.0, 120.0 (d, $^2J_{C-F}$ = 18.8 Hz), 116.0 (d, $^3J_{C-F}$ = 3.1 Hz), 112.1 (d, $^4J_{C-F}$ = 1.4 Hz), 30.7 (d, $^3J_{C-F}$ = 3.2 Hz). **^{19}F NMR ($CDCl_3$, 282 MHz):** δ = -175.6 (m). **HRMS (ESI):** calcd. for $C_{16}H_{11}O_3FNa$: m/z $[M+Na]^+$ 293.05844; found: 293.0586 (0 ppm).

Synthesis of 2-(3-fluoro-5-(prop-1-en-2-yl)benzofuran-2-yl)acetic acid 6

The reaction was performed with ester (60 mg, 0.24 mmol) and 1 mL LiOH according to the general procedure. After purification by chromatography on silica gel, acid **6** was obtained as a white solid (40 mg, 71% yield).

***R*f** = 0.42 (petroleum ether-EtOAc; 5:5); **m.p. =** 120-122°C.

^{1}H NMR ($CDCl_3$, 300 MHz): 7.61-7.58 (m, 1H),7.48-7.44 (m, 1H), 7.36-7.31 (m, 1H), 5.33-5.31 (m, 1H), 5.13-5.10 (m, 1H), 3.89 (d, 2H, *J* = 1.7 Hz), 2.22-2.19 (m, 3H). **^{13}C NMR ($CDCl_3$, 75 MHz):** δ = 174.3 (d, $^4J_{C-F}$ = 2.7 Hz), 151.3 (d, $^2J_{C-F}$ = 8.8 Hz), 146.2 (d, $^1J_{C-F}$ = 254.1 Hz, C_{11}), 143.0, 137.0, 133.1 (d, $^3J_{C-F}$ = 26.7 Hz), 123.4, 119.4 (d, $^2J_{C-F}$ = 18.8 Hz), 114.4 (d, $^3J_{C-F}$ = 3.1 Hz), 112.6, 111.5 (d, $^4J_{C-F}$ = 1.4 Hz), 30.8 (d, $^3J_{C-F}$ = 3.1 Hz), 22.2. **^{19}F NMR ($CDCl_3$, 282 MHz):** δ = -175.67 (m). **HRMS (ESI):** calcd. for $C_{13}H_{10}O_3F$: m/z $[M-H]^-$ 233.06195; found: 233.0618 (1 ppm); calcd. for $C_{12}H_{10}OF$: m/z $[M-CO_2-H]^-$189.07212; found: 189.0722 (0 ppm).

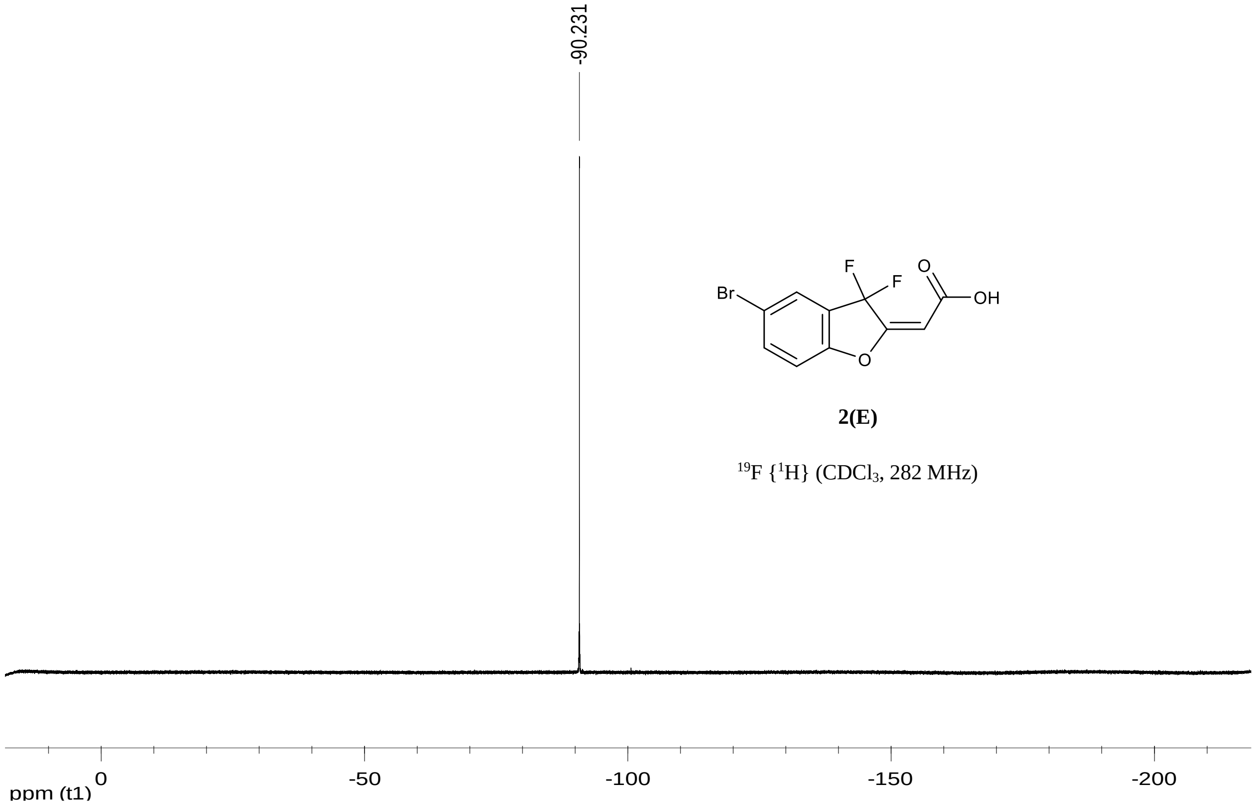
-90.231
Br
F
F
O
OH
O
2(E)
19F {1H} (CDCl3, 282 MHz)
ppm (t1)
0
-50
-100
-150
-200

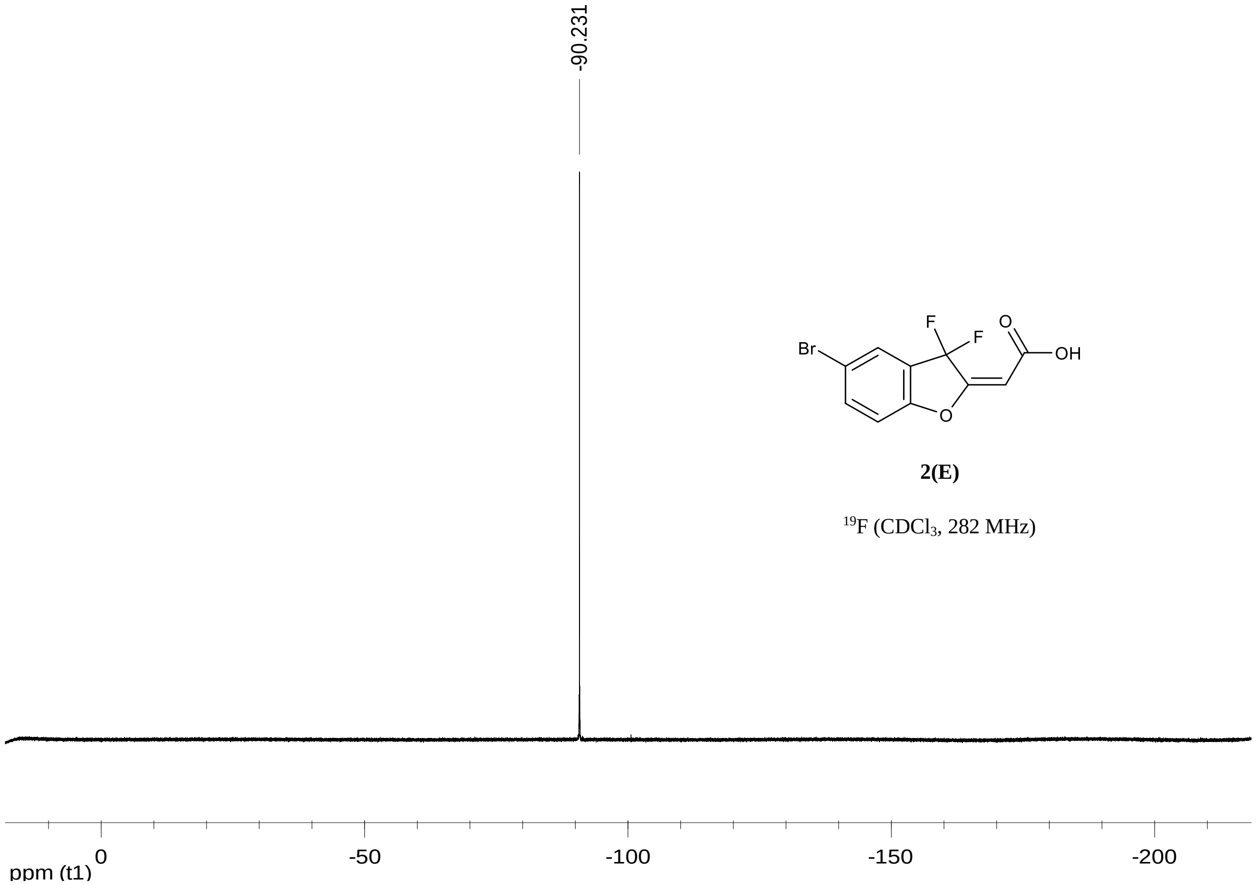
-90.231
Br
F
F
O
OH
O
2(E)
19F (CDCl3, 282 MHz)
ppm (t1)
0
-50
-100
-150
-200

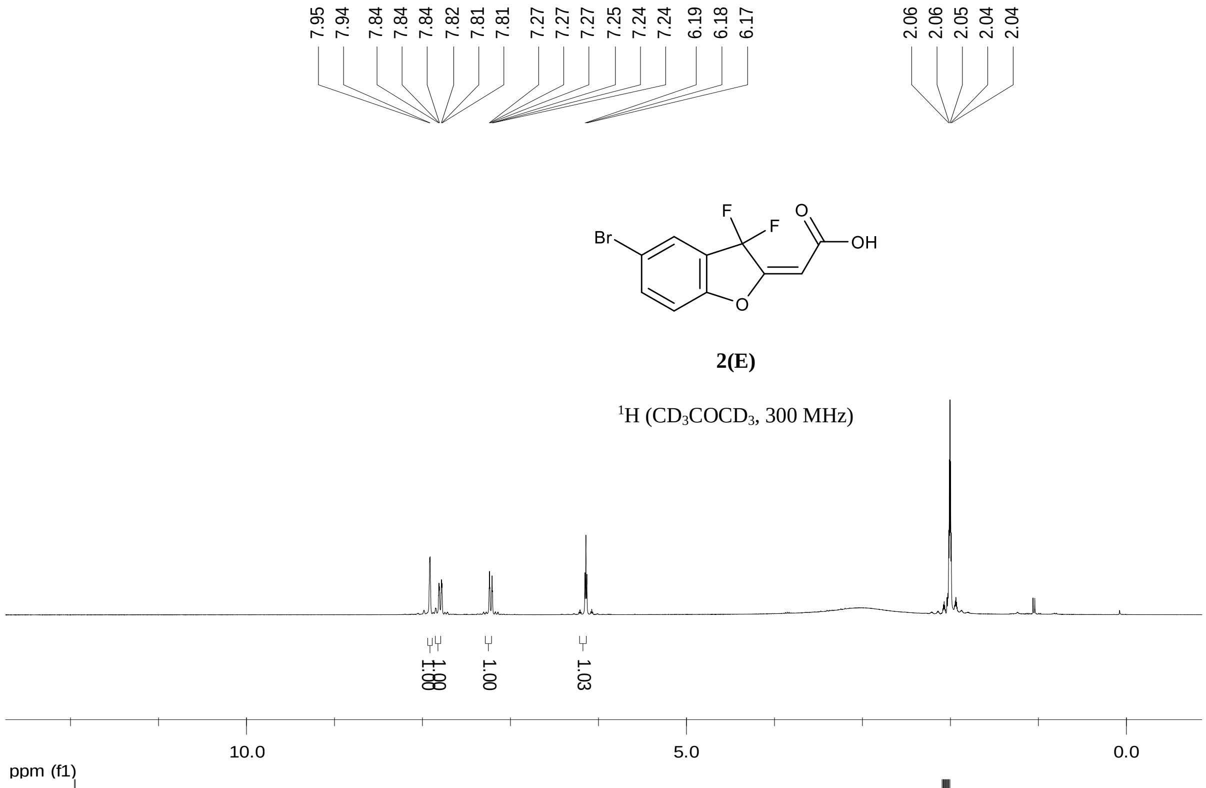

2(E)

^{1}H (CD_3COCD_3, 300 MHz)

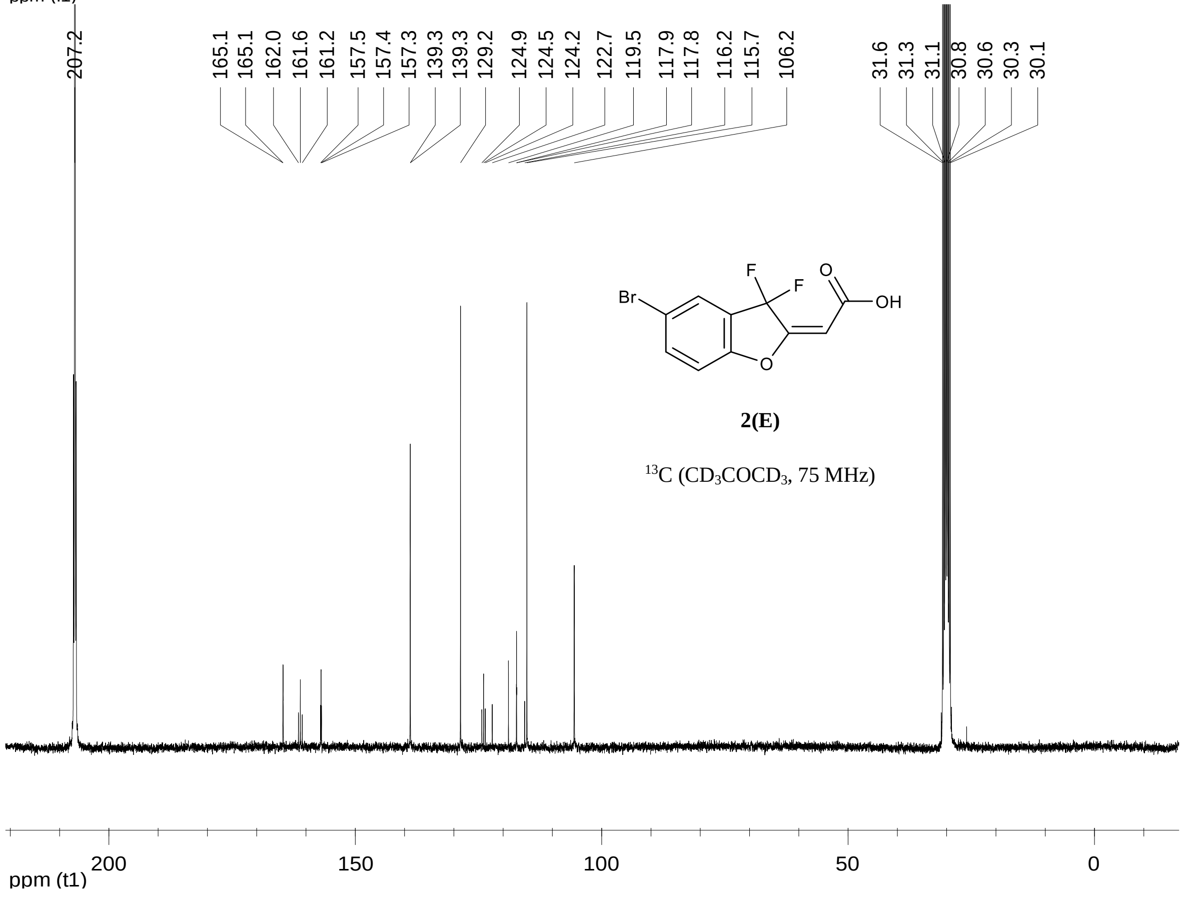

2(E)

^{13}C (CD_3COCD_3, 75 MHz)

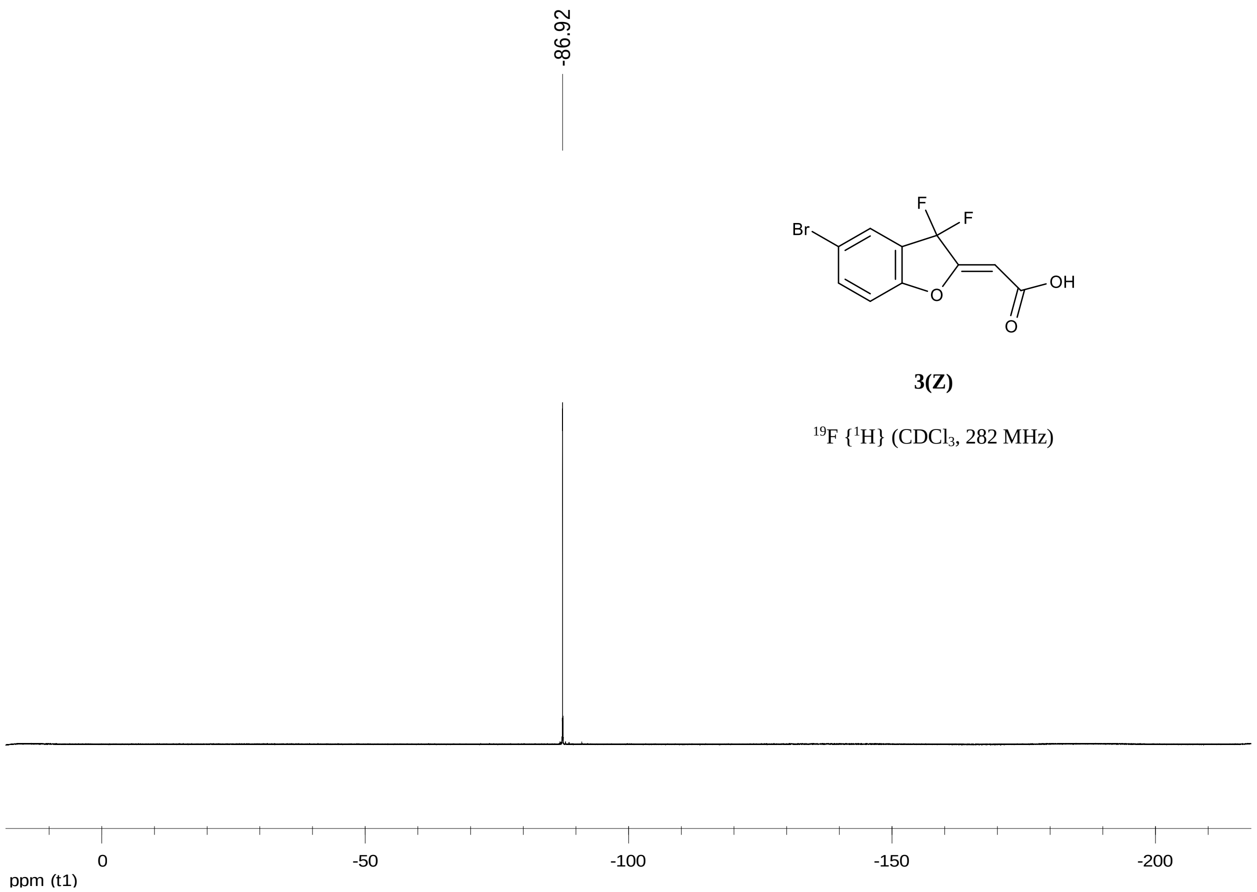
-86.92
Br
F
F
O
OH
O
3(Z)
^{19}F {^{1}H} ($CDCl_3$, 282 MHz)
0
-50
-100
-150
-200
ppm (t1)

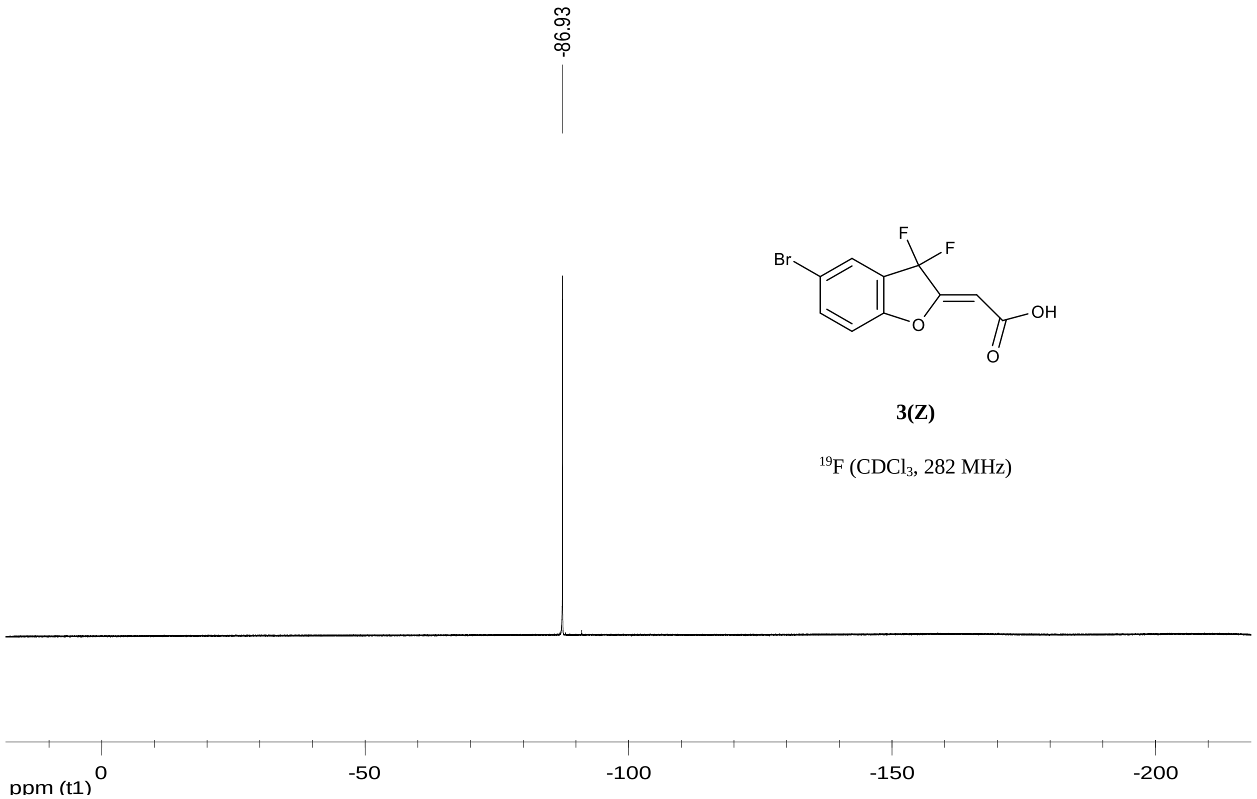
-86.93
Br
F
F
O
OH
O
3(Z)
^{19}F ($CDCl_3$, 282 MHz)
0
-50
-100
-150
-200
ppm (t1)

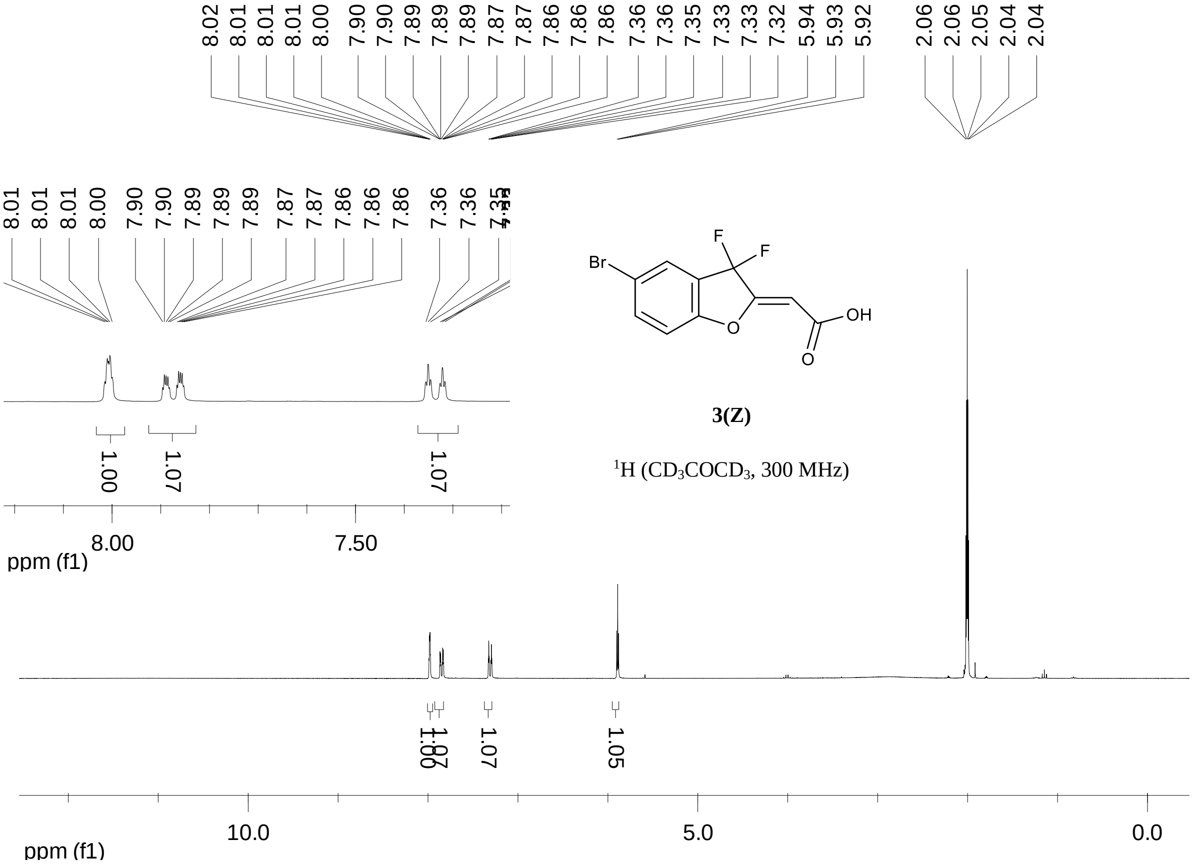
8.02 8.01 8.01 8.01 8.00 7.90 7.90 7.89 7.89 7.89 7.87 7.87 7.86 7.86 7.86 7.36 7.36 7.35 7.33 7.33 7.32 5.94 5.93 5.92 2.06 2.06 2.05 2.04 2.04
Br
F
F
O
OH
O
3(Z)
^{1}H (CD_3COCD_3, 300 MHz)
1.00
1.07
1.07
1.05
8.00
7.50
ppm (f1)
10.0
5.0
0.0
ppm (f1)

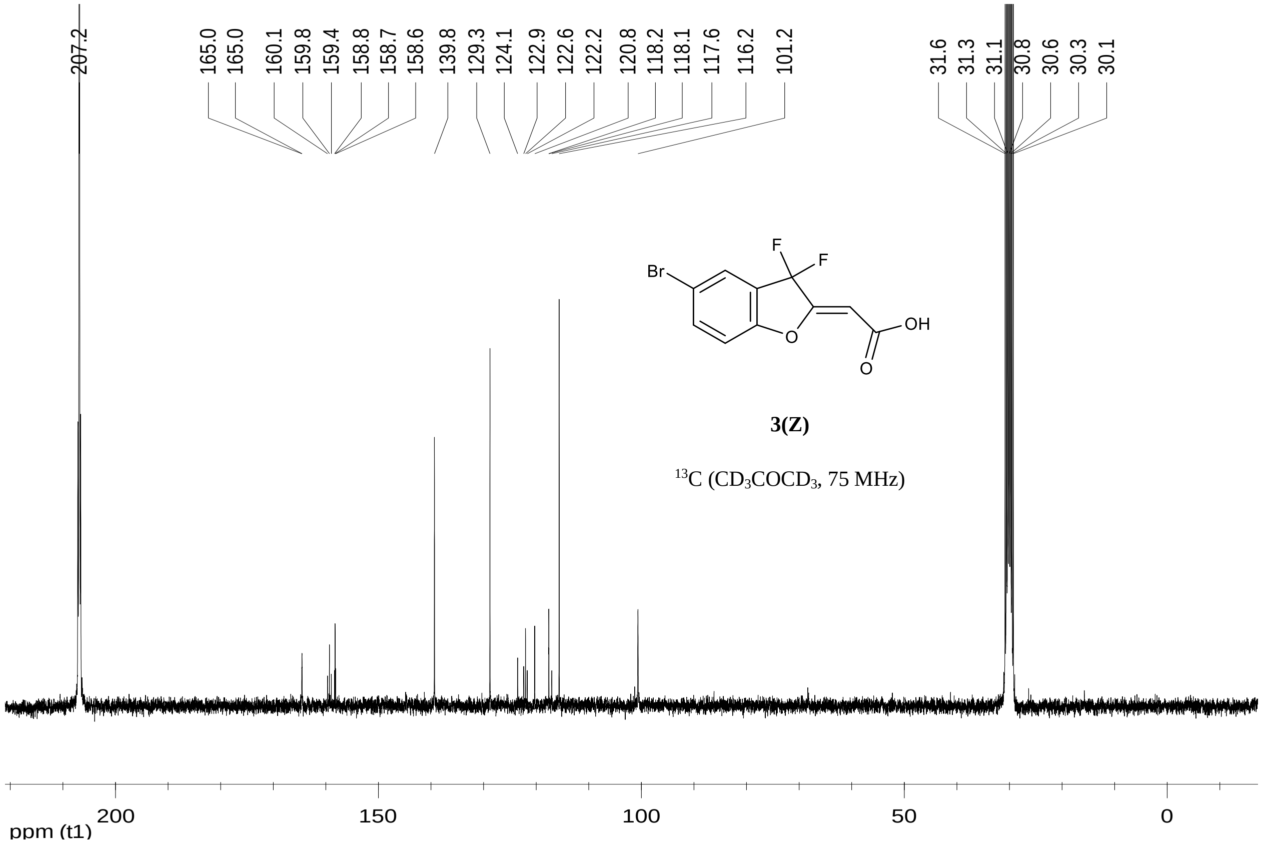
207.2
165.0 165.0 160.1 159.8 159.4 158.8 158.7 158.6 139.8 129.3 124.1 122.9 122.6 122.2 120.8 118.2 118.1 117.6 116.2 101.2
31.6 31.3 31.1 30.8 30.6 30.3 30.1
Br
F
F
O
OH
O
3(Z)
^{13}C (CD_3COCD_3, 75 MHz)
200
150
100
50
0
ppm (t1)

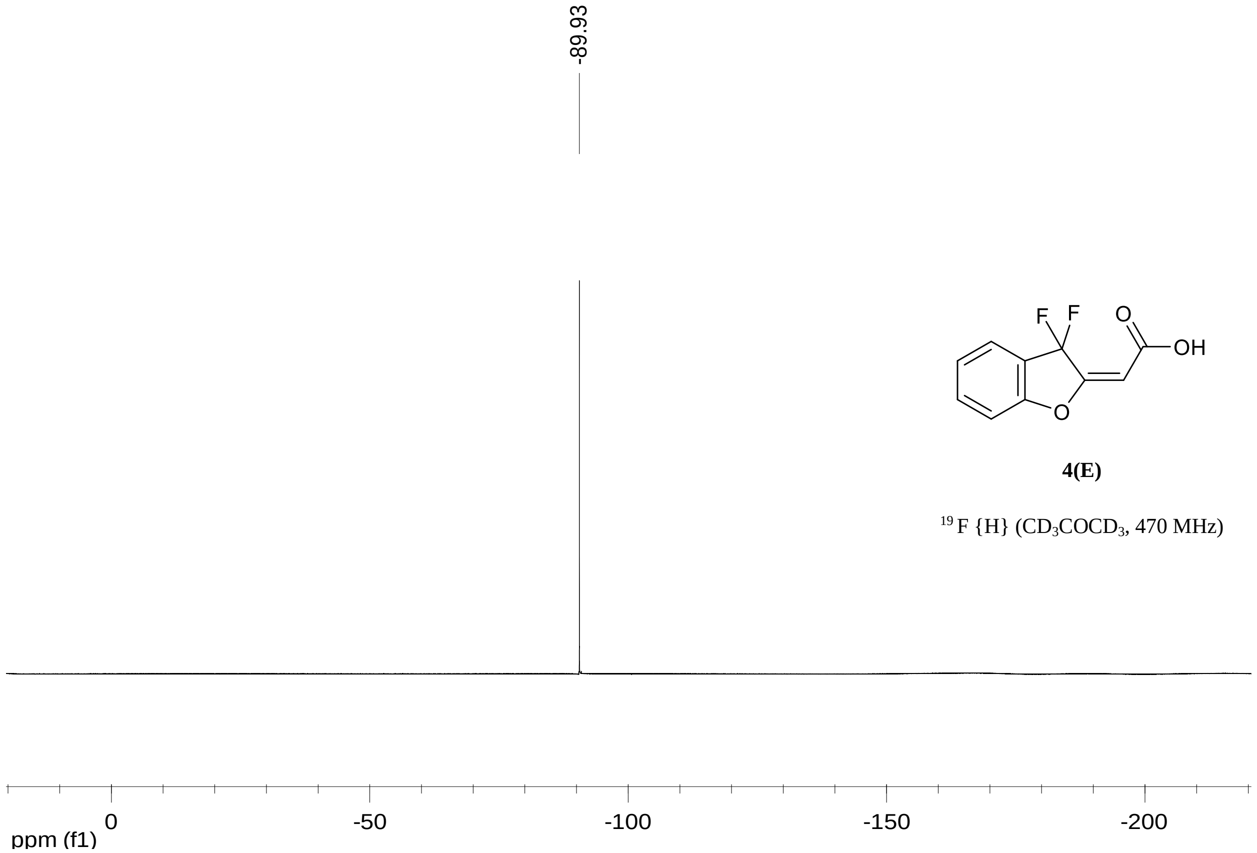
-89.93
F F O
OH
O
4(E)
^{19}F {H} (CD_3COCD_3, 470 MHz)
0
-50
-100
-150
-200
ppm (f1)

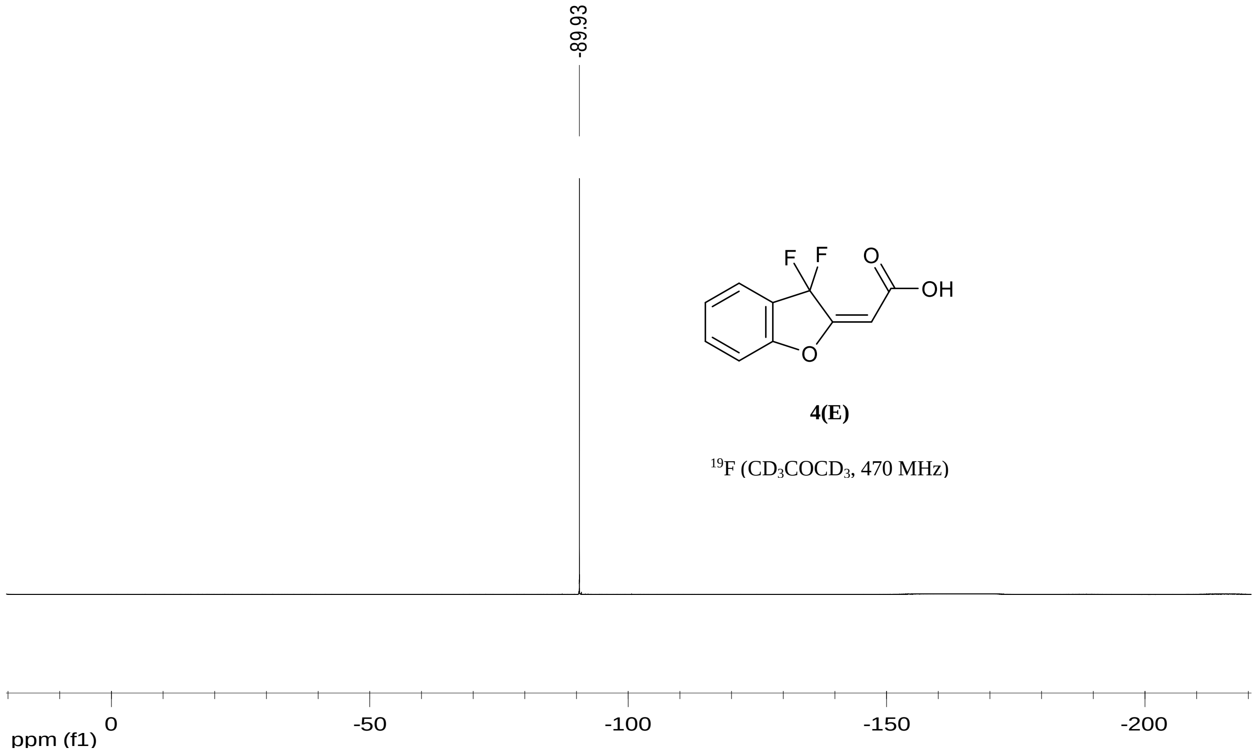
-89.93
F F O
OH
O
4(E)
^{19}F (CD_3COCD_3, 470 MHz)
0
-50
-100
-150
-200
ppm (f1)

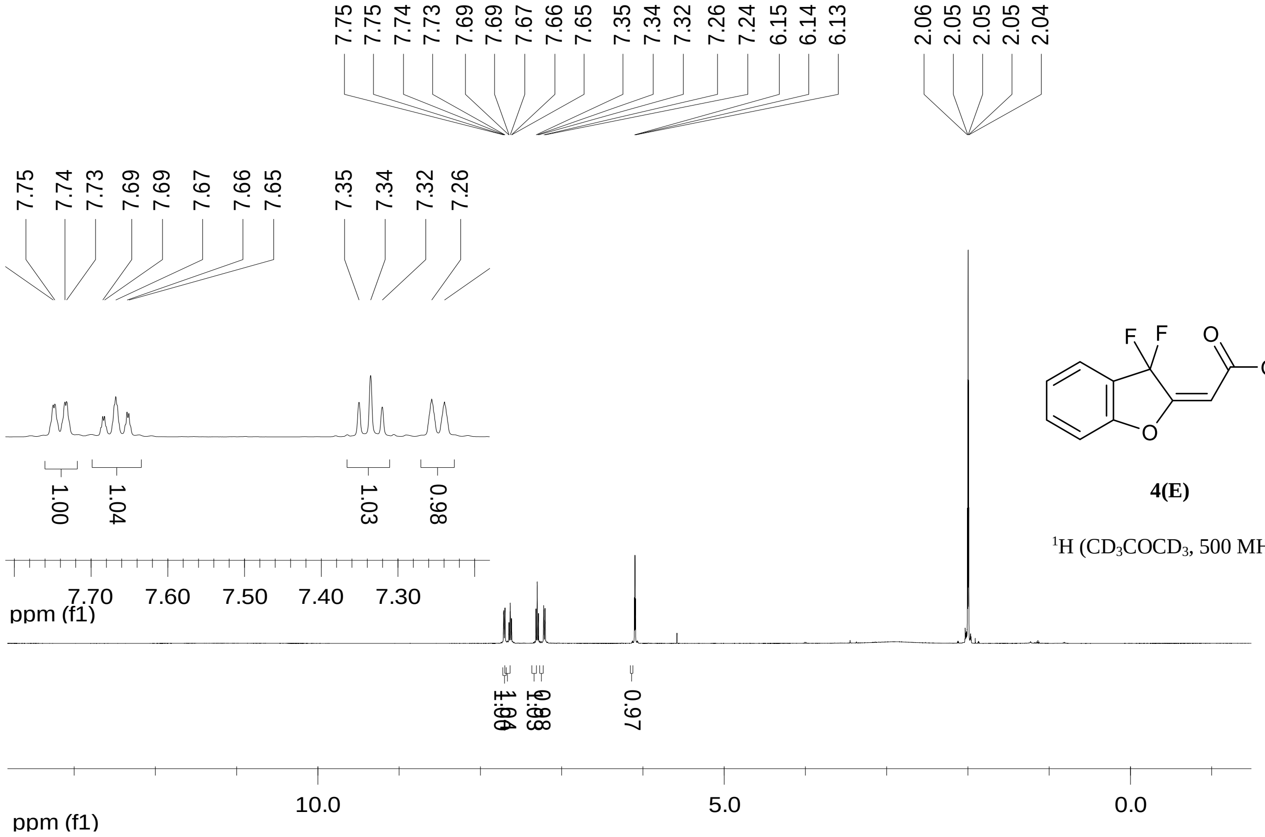

4(E)

^{1}H (CD_3COCD_3, 500 MHz)

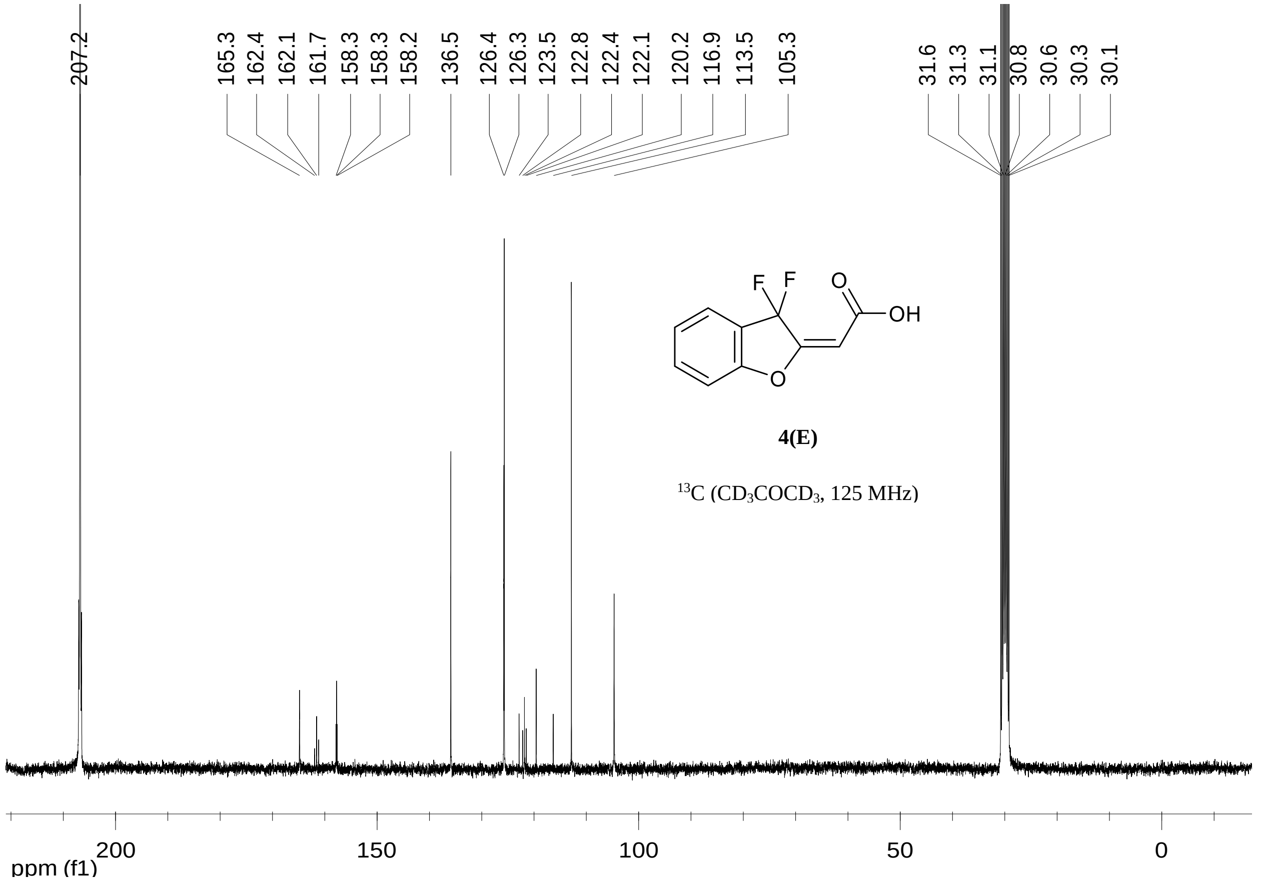

4(E)

^{13}C (CD_3COCD_3, 125 MHz)

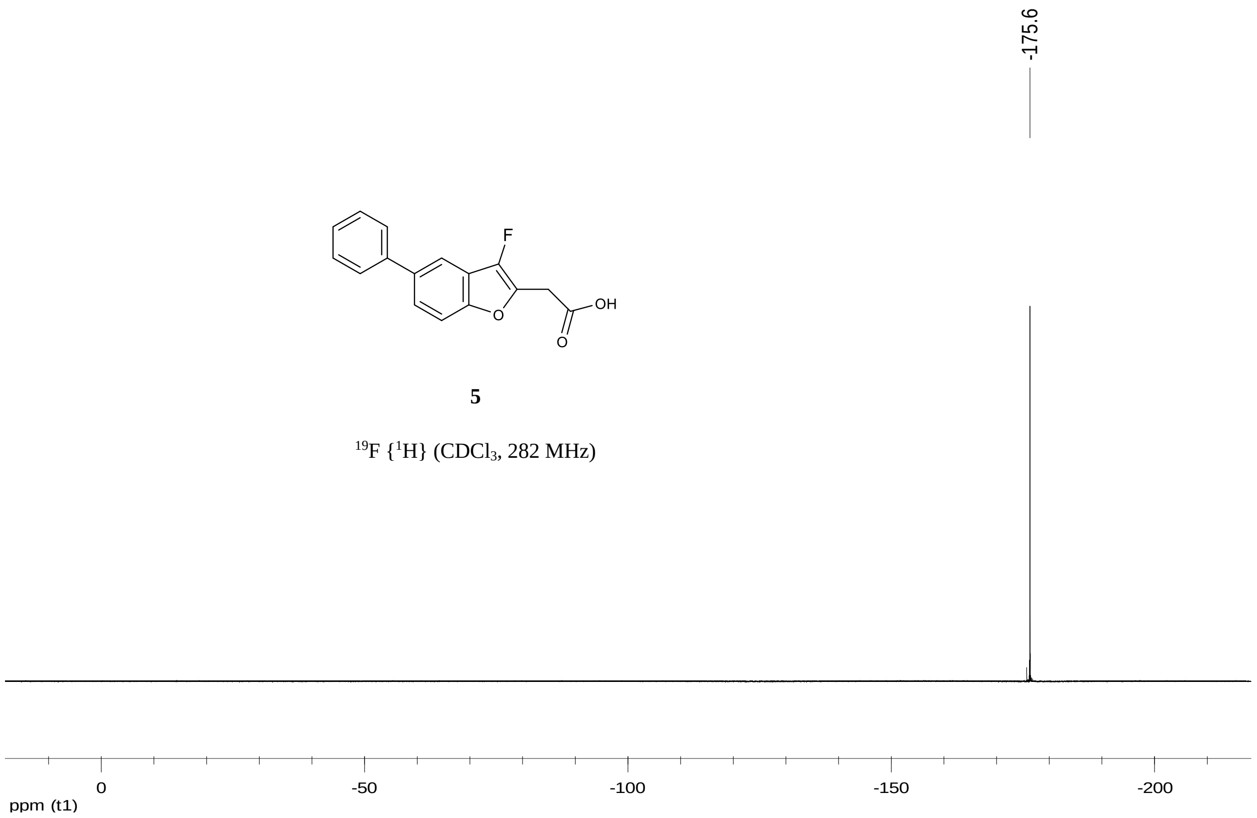
-175.6
F
OH
O
O
5
19F {1H} (CDCl3, 282 MHz)
0
-50
-100
-150
-200
ppm (t1)

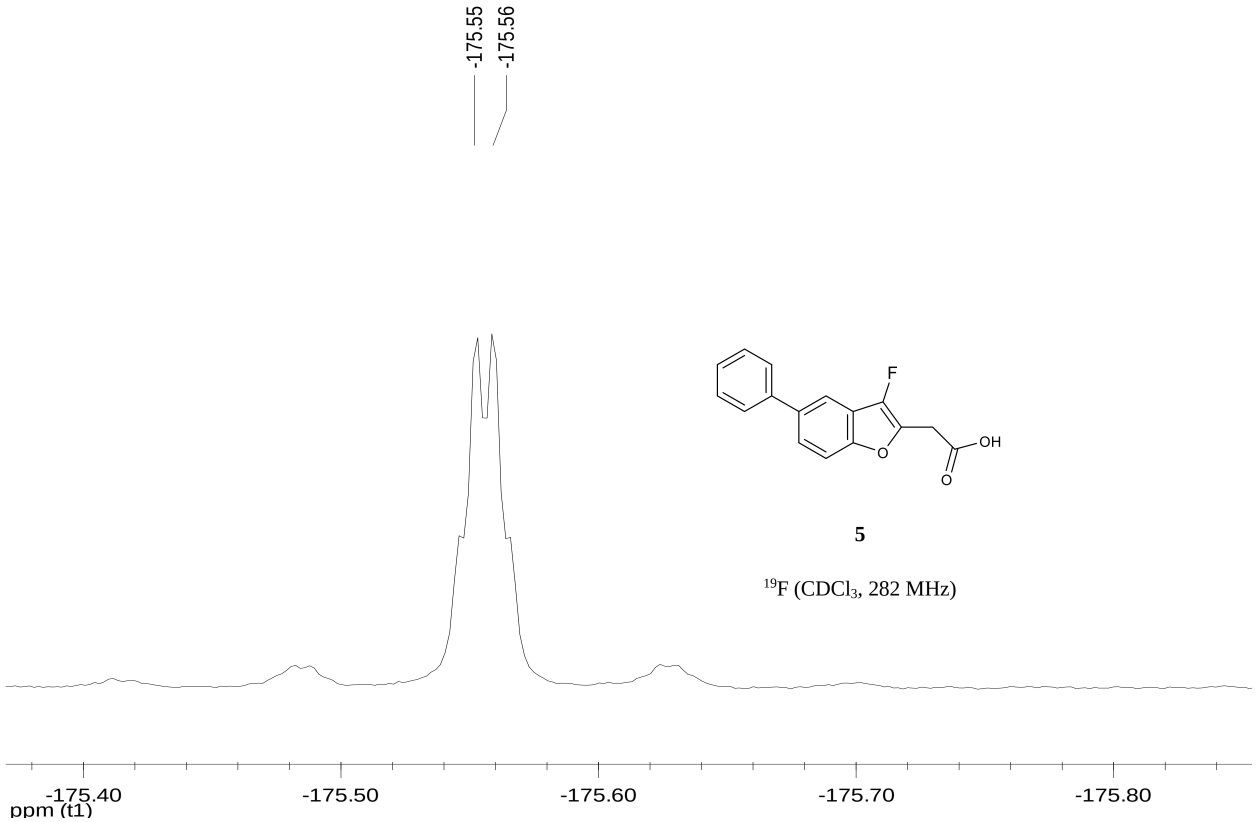
-175.55
-175.56
F
OH
O
O
5
19F (CDCl3, 282 MHz)
-175.40
-175.50
-175.60
-175.70
-175.80
ppm (t1)

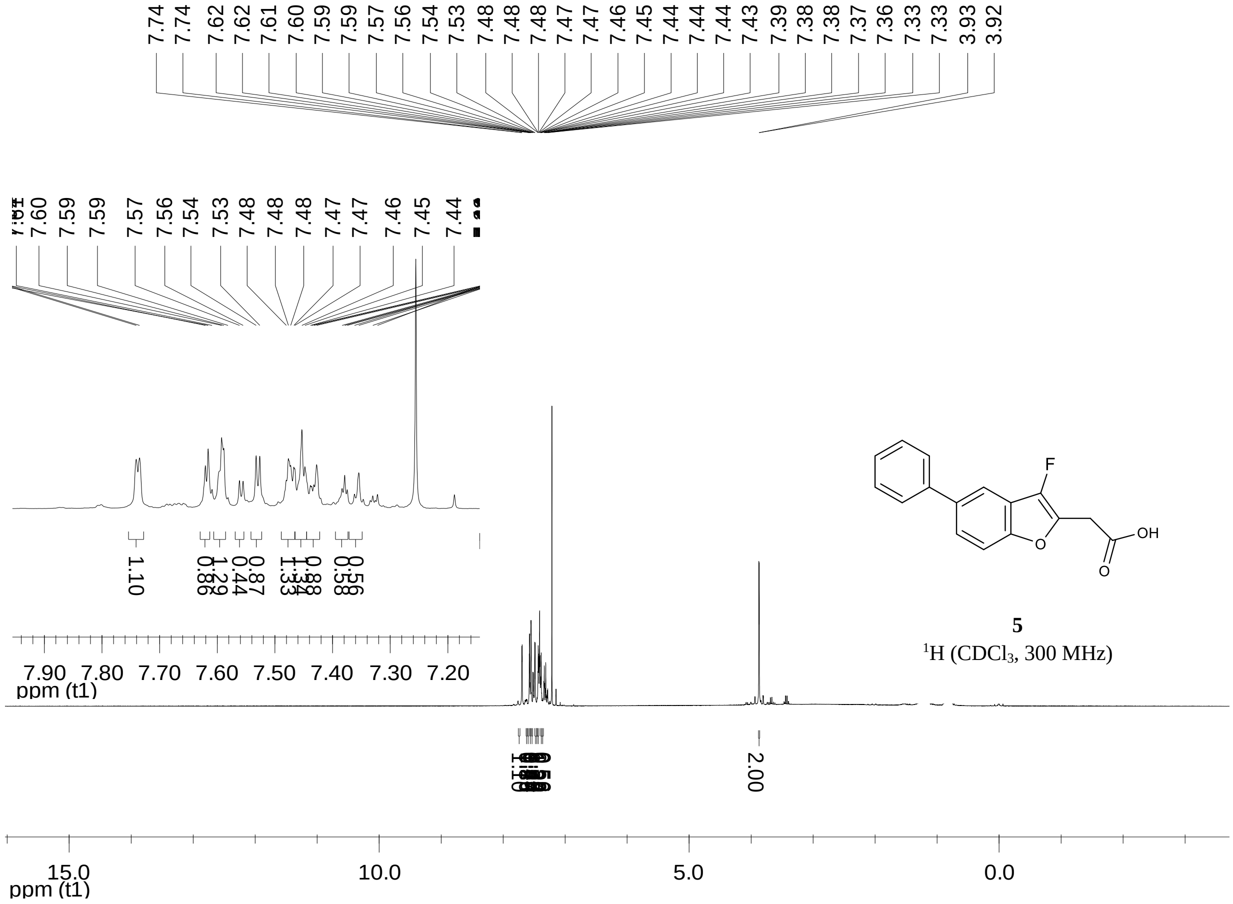

5

^{1}H ($CDCl_3$, 300 MHz)

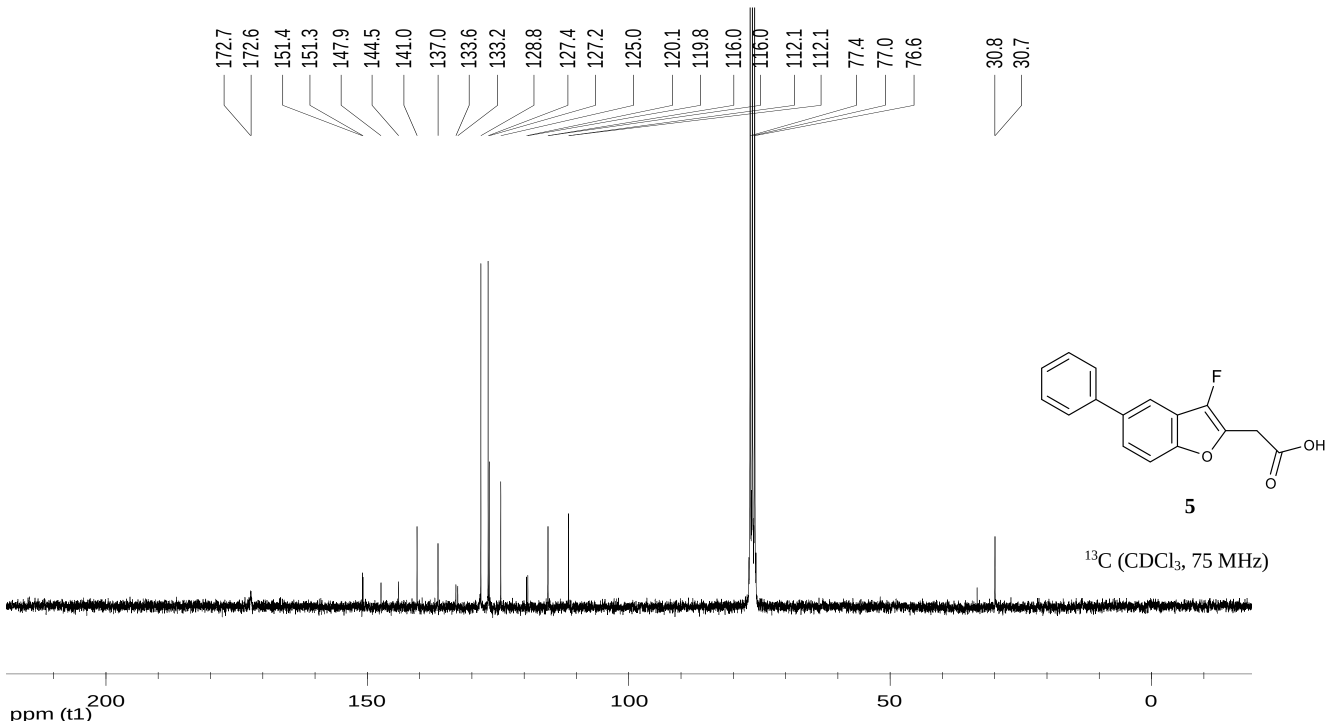

5

^{13}C ($CDCl_3$, 75 MHz)

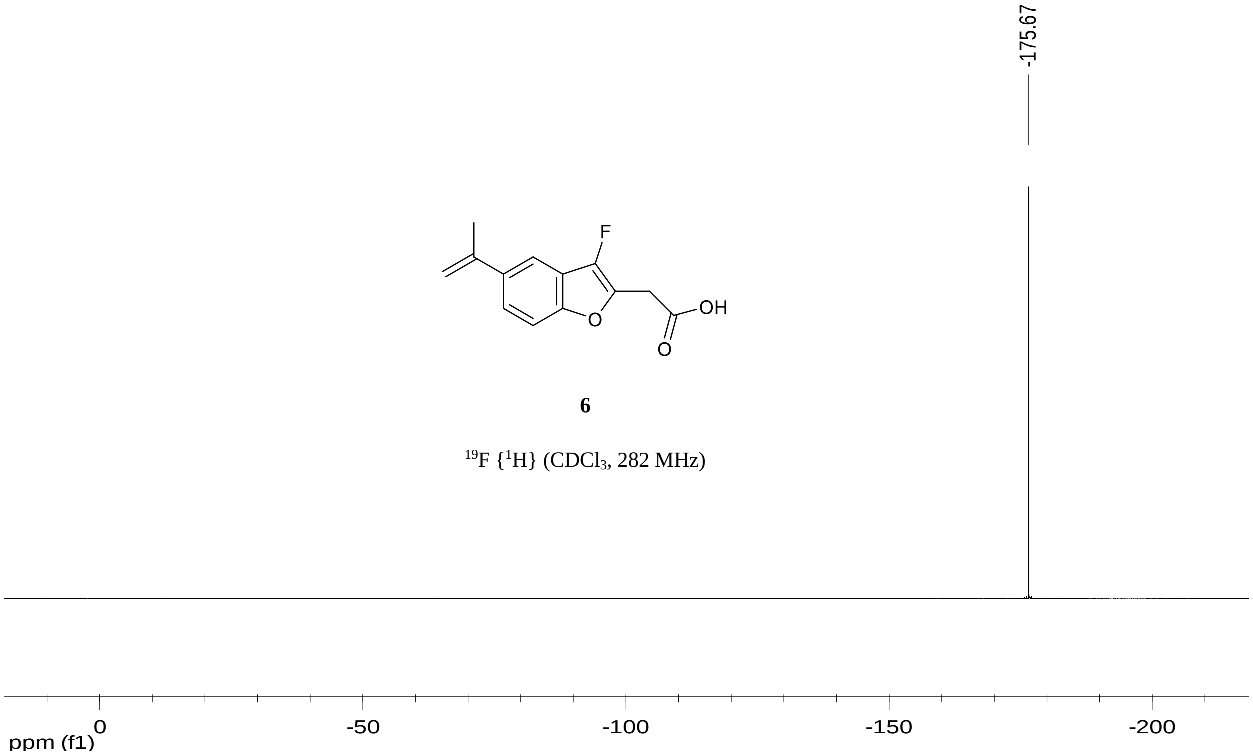
-175.67
F
OH
O
O
6
19F {1H} (CDCl3, 282 MHz)
0
-50
-100
-150
-200
ppm (f1)

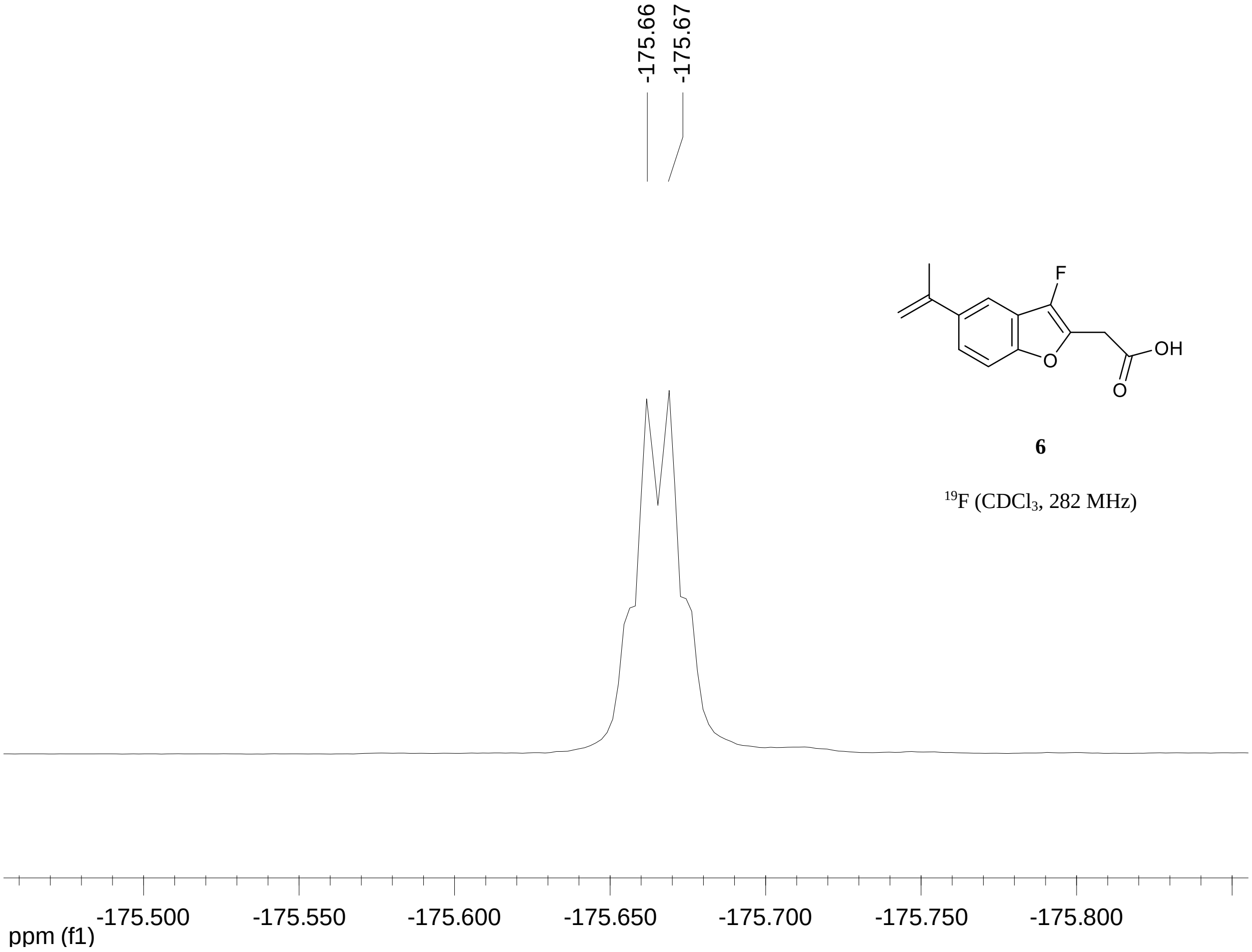
-175.66
-175.67
F
OH
O
O
6
19F (CDCl3, 282 MHz)
-175.500
-175.550
-175.600
-175.650
-175.700
-175.750
-175.800
ppm (f1)

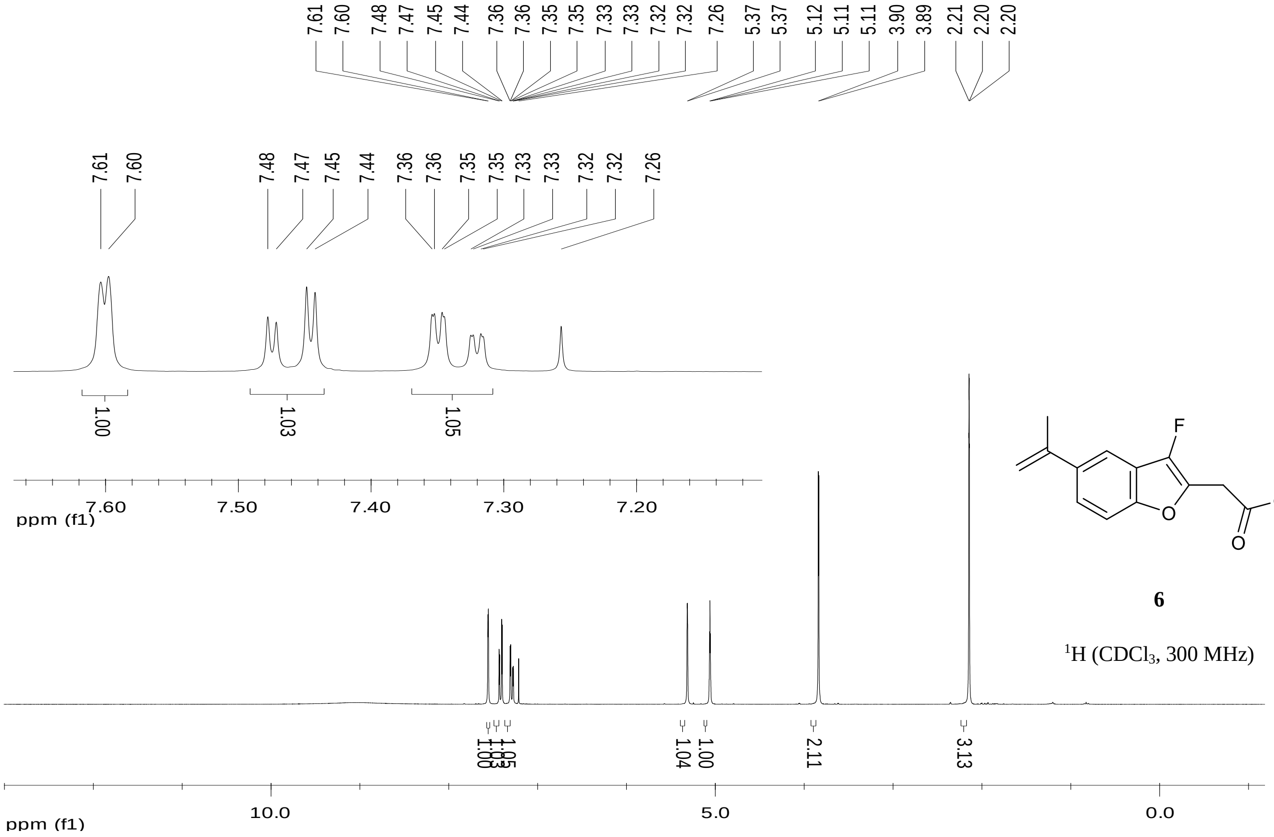
7.61 7.60 7.48 7.47 7.45 7.44 7.36 7.36 7.35 7.35 7.33 7.33 7.32 7.32 7.26 5.37 5.37 5.12 5.11 5.11 3.90 3.89 2.21 2.20 2.20
1.00 1.03 1.05
ppm (f1) 7.60 7.50 7.40 7.30 7.20
F
OH
O
O
6
^{1}H ($CDCl_3$, 300 MHz)
1.00 1.03 1.05 1.04 1.00 2.11 3.13
ppm (f1) 10.0 5.0 0.0

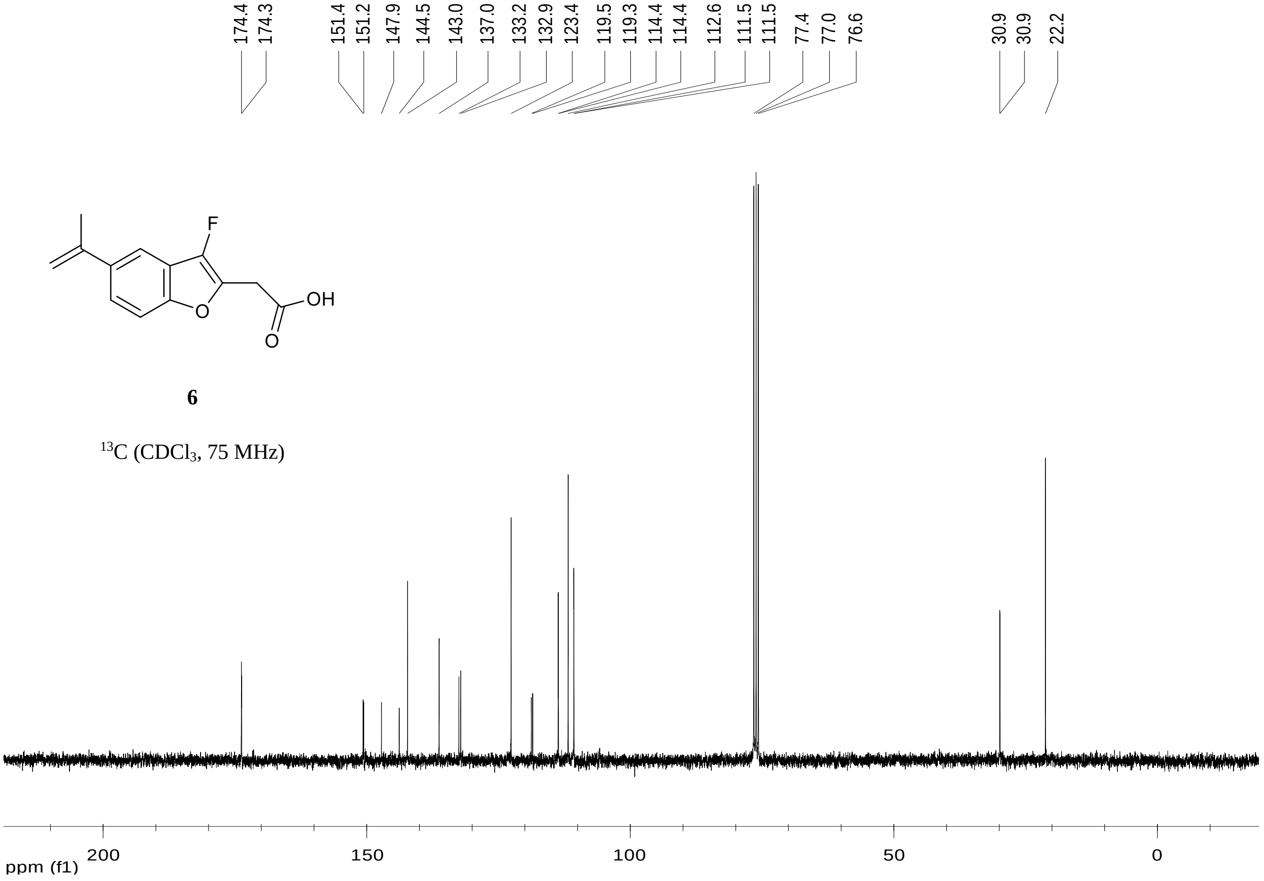
174.4 174.3 151.4 151.2 147.9 144.5 143.0 137.0 133.2 132.9 123.4 119.5 119.3 114.4 114.4 112.6 111.5 111.5 77.4 77.0 76.6 30.9 30.9 22.2
F
OH
O
O
6
^{13}C ($CDCl_3$, 75 MHz)
ppm (f1) 200 150 100 50 0
